# Modeling Singapore's First African Swine Fever Outbreak in Wild Boar Populations

**DOI:** 10.1155/2024/5546893

**Published:** 2024-08-26

**Authors:** Rayson Bock Hing Lim, Zhan Pei Heng, Kelvin Ho, Kane Koh, Hwee Ping Lim, Kelvin Lim, Wendy Sng, Gordon Tan, Ernest Teo, Tze Hoong Chua

**Affiliations:** Veterinary Health Division Animal and Veterinary Service National Parks Board, Singapore, Singapore

## Abstract

African swine fever (ASF) is a virulent and lethal disease affecting domestic pigs and wild boars, with serious implications for biodiversity, food security, and the economy. Since its reemergence in Europe, ASF has become widespread, and Singapore reported its first ASF outbreak in its wild boar population. To understand the transmission dynamics in Singapore's urban landscape, an agent-based spatiotemporal model was designed to mechanistically model the wild boar dispersal and their interactions for ASF transmission. We investigated the impacts of wild boar dispersal capacity and carcass removal actions on the spatiotemporal dynamics of disease transmission. The model predictions were validated using observed wild boar mortality reports in Singapore and suggested multiple disease entry points into our wild boar population. Our simulations estimated that the ASF outbreak in Singapore would peak within 3 weeks and lasts for less than 70 days. Carcass-mediated transmission was evident with epidemic reoccurrence through infectious carcasses accounting for 18%–75% of the iterations. Increasing wild boar dispersal capacity expanded the geographic extent of ASF infection, potentially spreading further inland. Simulated carcass removal and decontamination measures slightly reduced the epidemic duration by up to 13.5 days and reoccurrence through infectious carcass by 10.8%. Carcass removal and decontamination efforts, along with identifying and blocking high-risk areas (e.g., dispersal corridors), are important in controlling the transmission of ASF through contaminated fomites and limiting the dispersal of infected animals. Establishing surveillance programmes and enhancing detection capabilities are also crucial for the successful management and control of infectious diseases.

## 1. Introduction

African swine fever (ASF) is a highly contagious and deadly disease affecting domestic pigs and wild boars (e.g., *Sus scrofa*) worldwide [1]. It is caused by the ASF virus (ASFV), a double-stranded DNA virus from the family *Asfarviridae*, and is lethal for many domestic and wild pig species [2]. The disease was first recognized to be caused by ASFV in 1910, when domestic pigs were introduced into Kenya during the colonial era [3]. Subsequently, it was revealed that ASFV is naturally maintained within sub-Saharan Africa through sylvatic circulation between the Argasid soft ticks (*Ornithodoros* spp.) as the vector, and wild warthogs (*Phacochoerus africanus*) and bush pigs (*Potamochoerus larvattus*) as the reservoir host, which do not succumb to the lethal effects of the infection [3, 4, 5]. Since then, ASF outbreaks have been reported in Africa and Europe, although the outbreaks were successfully contained and eradicated in most instances before 2007 [3].

ASFV is highly transmissible among pigs through direct (oral, blood, and scavenging) and indirect (fomites, vector, and excretion) contact with reported mortality rate of up to 100% [6, 7]. Convalescent carriers (i.e., infectious survivors) and carcass-mediated infection (i.e., scavenging) are also important transmission mechanisms that can influence ASFV persistence in the blood and tissues of surviving pigs and the environment [8]. Extended viral persistence in the environment may lead to delayed transmission in the population, thereby increasing the chance for reintroduction of the disease, leading to recurrent epidemics [8].

In the last two decades (2000–2020), ASF outbreaks resulting from a single introduction of the Southeast African genotype II ASFC isolated from Georgia in 2007 [9] spread rapidly to neighbouring countries (Azerbaijan and Armenia) and northward into Europe and Russia [1, 10, 11]. The spread of ASF has accelerated since 2018, affecting numerous countries including China (2018–2020), South Korea (2019–2022), Laos (2019), Vietnam (2020), and Malaysia (2021–2023) [12]. The prolonged global ASF outbreaks resulted in the deaths of more than 100 million domestic pigs, devastating the pork industry [13] and decimating wild pig populations globally with serious implications on their conservation, food security, and economy [2].

On 5^th^ February 2023, Singapore reported its first ASF case from a wild boar carcass found in a nature park [14]. Singapore does not have any domestic pig production, and free-living wild boars in parks and nature reserves as well as suids reared in captivity within wildlife parks represent the only susceptible populations to ASF. The current practice for the monitoring and management of ASF in Singapore's wild boars is to enhance biosurveillance for active detection, trapping of infected wild boars, reporting carcasses to be removed, and decontamination of the infected area. Since then, a total of 49 wild boar carcasses were reported between February and March 2023, of which 18 tested positive for ASF. The remaining reports were negative or considered to be inconclusive as the carcasses were badly decomposed. Most of the carcasses were confined within the western part of mainland Singapore and Pulau Ubin, an offshore island located in the northeast of Singapore. While the source of the ASF outbreak in Singapore is unknown, we postulate that the disease was likely introduced through infected wild boars that crossed Singapore's border from affected regions, and this coincided with reports from the region during the same period [15, 16].

The threat posed by ASF to wild boars and domestic pigs is significant [13], and understanding the mechanistic processes affecting the epidemiology of the disease is critical to predict and control the transmission of diseases in affected populations [17]. The epidemiology of ASF has been extensively studied in Russia and European countries using simulation models [8, 10, 18, 19]. These simulations and models are useful for the predicting and understanding of the ASF transmission dynamics, and evaluating the efficacy of various management responses (e.g., preemptive depopulation, enhanced surveillance programme, contact tracing, and biosecurity) to control the spread of ASF [18, 20, 21, 22]. However, the lack of targeted research and surveillance capacity on ASF transmission among free-ranging wild boars in Southeast Asia suggests that the extent and prevalence of the disease circulating among wild boar populations in the region remain unclear [2, 23].

In this study, we aim to investigate the fine-scale spatiotemporal transmission and persistence of ASF among wild boars in Singapore using an agent-based model that considers direct boar-to-boar and carcass-mediated transmissions, the habitat-dependent movement behaviour, and wild boar population densities in Singapore. The model fit was evaluated by comparing model predictions to wild boar mortality reports which included confirmed cases infected with ASFV in Singapore. We also evaluated the effects of active management measures (i.e., active carcass removal and decontamination efforts) and the dispersal capacity of wild boars on the ASF disease dynamics in Singapore.

## 2. Materials and Methods

### 2.1. Model Overview

We developed an agent-based approach to examine the spatiotemporal transmission dynamics of ASF in Singapore's wild boar population by simulating the boar-to-boar interactions across a real-world landscape. In this study, wild boars were distinguished by their health state (susceptible, exposed, infectious, dead, and decomposed) and the agents exhibited stochastic habitat-dependent movement behaviour to emulate the movement dynamics of wild boars in Singapore. The disease transmission among agents depends on nearest-neighbour contact, habitat-dependent movement, the stochastic probability for transmission (i.e., exposure and infection) and mortality, as well as time-dependent epidemiological parameters (e.g., latent period) of ASF. These processes will be detailed below.

### 2.2. Study Site and Landscape Structure

**Figure 1:**
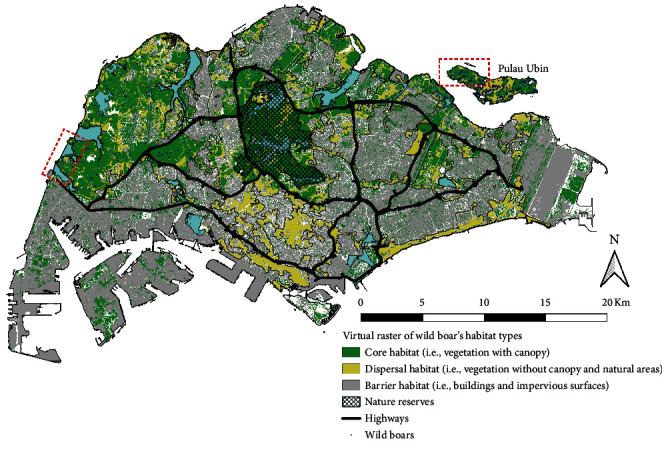
Map of Singapore with the presumed habitat types (core, dispersal, and barrier) used for the simulation. The hypothesized disease landing zones are indicated on the map with a red box.

Singapore is a highly urbanized city–state, with a total land area of 742.2 km^2^, consisting of a complex mosaic of various land use types: vegetation (46.6 km^2^), buildings (284.1 km^2^), and pervious surfaces [24]. Singapore's forested natural areas on the mainland are mostly restricted to parks, nature reserves, and areas located in the central and western part of the mainland (Figure 1). The small offshore island, Pulau Ubin, is largely uninhabited and undeveloped with a large wild boar population [15].

In our simulation, the virtual landscape (Figure 1) was created using land-use data based on a high-resolution terrestrial map of Singapore [24]. Using published information on wild boar habitat association and genetic connectivity in Singapore, we identified the wild boar core habitat as forested areas (i.e., vegetated areas), and dispersal habitat as open and seminatural areas (i.e., nonvegetated pervious, marshes, swamps, and mangroves) [25, 26, 27, 28, 29]. Urban built-up areas and road network (i.e., buildings and impervious surfaces) as well as inland and coastal water bodies (i.e., reservoirs and open sea) were identified as barrier habitats that limit wild boar movement [18, 30, 31, 32]. We assumed that infected wild boars were unable to disperse across large water bodies owing to the lethality of ASFV. To manage computation cost yet allowing effective representation of Singapore's land-use gradient, we aggregated and reclassified the land-use data to 100 m^2^ cells for Singapore (total of 230,505 cells) using the raster package [33] in R version 4.0.3 [34]. Within the aggregated cell, we calculated the proportion of core, dispersal, and barrier habitat types, where the habitat type (i.e., core, dispersal, or barrier) with the highest proportion will be assigned to the aggregated cell.

### 2.3. Individuals: Population Density and Distribution

Wild boar (*S. scrofa*) was formerly thought to be extinct on mainland Singapore, with small populations found only on two offshore islands in Singapore [35]. Within the last two decades, wild boars have successfully recolonized the main island, possibly dispersed from Peninsular Malaysia and are now widespread throughout Singapore [15, 35]. We extrapolated published information on the wild boar density in the Central Catchment Nature Reserve (CCNR) [15, 28, 36] across Singapore to estimate the total wild boar population (*Supplementary 1*). The spatial distribution of the wild boars was represented in our model by randomly allocating each wild boar to one core habitat cell while ensuring that the distribution is within the reported mean density estimated for mainland Singapore and Pulau Ubin (*Supplementary 1*).

### 2.4. Movement: Decision and Distance

The dispersal behaviour and movement pattern of wild boars are influenced by their foraging and resting activities as well as the underlying habitat [37, 38]. In Singapore, previous studies on their movement ecology suggested small activity ranges (0.89 km^2^; [36]) with peak foraging activity occurring at dusk (1800 hr; [28]). Wild boar presence was also positively associated with natural habitats such as primary and secondary forests, but negatively associated with open areas and roads [28]. However, other studies reported that wild boars travel long distances with home ranges of 4–11 km^2^ [36, 37, 39, 40] and distance traveled ranging between 1.7 and 12.9 km per day [41, 42, 43, 44, 45]. To comprehensively emulate the agent's movement behaviour over the possible dispersal ranges (2–8 km), we used a Bernoulli process to determine the habitat-dependent movement decision of each agent. Specifically, the agent's decision to move (*p*_move_) is calculated as:

(1) $$\begin{array}{c} p_{\text{move}}=1-p_{\text{Habitat}\_\text{use}}, \end{array}$$

where *p*_Habitat_use_ is the probability that the wild boar will utilize the habitat it is currently inhabiting. This assumes that the greater the habitat suitability (i.e., for foraging or resting), the less inclined the agent will relocate. Hence, when the agent inhabits a dispersal habitat (i.e., open and seminatural areas), there is a greater tendency for the wild boar to continue moving until they occupy a core habitat where they will spend more time foraging, resting, or seeking refuge.

The temporal resolution of our model was calibrated using movement simulations in a disease-free setting (*Supplementary 2*). Specifically, an agent was randomly assigned to a core habitat, and its movement and position were tracked for a total of 3,000 time-steps. At each time-step, the cumulative Euclidean distance traveled was calculated and compiled for 100 iterations. The number of time-steps required to achieve the predefined dispersal distances (2, 4, 6, and 8 km) was estimated through linear regression. This corresponds to temporal resolution of 60, 110, 165, and 215 time-steps, which equates to 24, 13, 9, and 7 min per time-step, respectively (Table 1).

### 2.5. Disease Transmission

**Figure 2:**
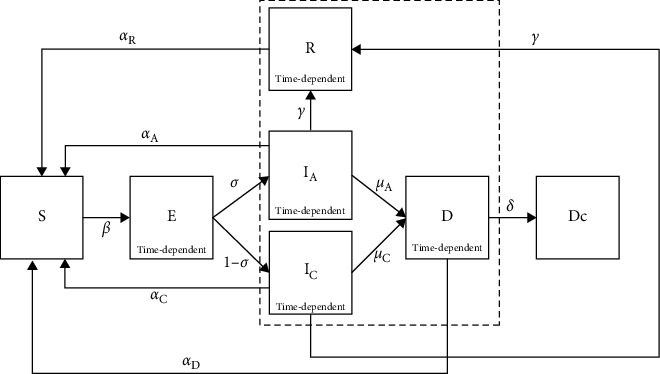
Overview of the ASF transmission dynamics. The agents are divided into various compartments: susceptible (S), exposed (E), infected (I_A_ and I_C_), recovered (R), dead (D), and decomposed (Dc). The infectious agents are demarcated within the dotted box. The transitions from exposed → infected, infected → recovered, infected → dead, and dead → decomposed are time-dependent processes.

We modified the SEIR epidemiological model to include the effects of carcass-mediated and convalescent carrier transmissions. The agents can be compartmentalized into six health states—susceptible (S), exposed (E), infected (I), recovered (R), dead (D), and decomposed (Dc; Figure 2). The transitions across states are stochastic processes that depend on the agents' interactions and time (i.e., latent period, infection duration, time to death, and recovery time). In our simulation, a susceptible individual transits to the exposed state with a mean probability *β* drawn from a Bernoulli distribution when it interacts with an infected, dead, or recovered agent. We assumed that recovered individuals are convalescent carriers as studies have shown that recovered pigs remain viraemic although being far less infectious than in the acute phase of infection [48]. We specified different transmission probabilities for infected (*β*_I_), recovered (*β*_R_), and dead (*β*_D_) agents to account for differences in virus-shedding dynamics through nasal, oral, excretion, and blood routes [49]. Exposed agents will only transit to the infected state after the mean latent period (*λ*) that is drawn from a Poisson distribution. We dissociated the infected agents into two substates: acute (IA) and chronic (Ic) substates which differentiated based on the severity parameter (*σ*) and characterized with different mortality rates (*μ*_A_ and *μ*_C_) and average time from infection to death for each substate. The infected agent either dies with a mean probability (*μ*_A_and *μ*_C_) and remains infectious in the environment until it completely decomposes after a specified period [18], or recovers with a mean probability (*γ*) and becomes a convalescent carrier and is immune to infections during the recovery period [50].

### 2.6. Model Parameters

Bayesian inference using sequential Monte Carlo (SMC) algorithm following procedures from Le et al. [51] was adapted to estimate certain model parameters (Table 2). The SMC algorithm involved sampling all combinations of the parameter values and sequentially reweighting the values using a log-likelihood function [58]. By seeking to minimize the difference between observed data (i.e., mortality reports related to ASF) and simulated outputs, it can provide an unbiased estimate of the posterior distribution for the set of unknown parameters more efficiently than the standard Markov chain Monte Carlo (MCMC) approaches [58]. Considering the nature of the parameters used in the simulation, we restricted the bounds of the prior distributions for the model parameters to the range of plausible values identified from published literature (Table 2). The SMC algorithm was coded in R, and details on the prior and posterior distributions can be found in *Supplementary 3* and *4*. The carcass persistence values used in our model were inferred using a range of carcass decomposition rates and virus persistence values reported in various literatures and adjusted for the tropical climate [7, 15, 55, 56]. The finalized parameter values used in our simulation are presented in Table 2.

### 2.7. Process Scheduling

At each discrete time-step, the agents follow the procedures in the given order: movement, pathogen transmission to neighbours, manifestation of symptoms, recovery, death, and decomposition. Each simulation was performed for up to 150 days or until the disease has been eliminated from the population (i.e., no more infectious or carrier agents). Each iteration was initialized with one infected agent occurring at random at our hypothesized disease landing zones (Figure 1). Under the active management scenario, we shortened the time of carcass persistence to 3 days to represent active management efforts for carcass detection and removal. A total of 800 unique iterations were performed for all combinations of management efforts (e.g., active and passive) and dispersal distance (2, 4, 6, and 8 km) in this study.

### 2.8. Analysis

The simulation and virtual landscape were programmed in [34]. The total number of exposed, infected, and dead agents were recorded, and the spatial distribution of the agents were tracked daily during the simulation. From these records, summary statistics of the population dynamics, their health status, the spatial distribution of the disease, and epidemic duration (days) were calculated for wild boars on mainland Singapore and Pulau Ubin. The maximal geographical extent of the infected area on mainland Singapore and Pulau Ubin were calculated based on the convex hull defined by the locations of all carcasses. Based on the geographical extent of the infected area for each simulation, we calculated the overall landscape contiguity of core and dispersal habitat types using the landscapemetrics package [59] in R. The codes for the agent-based simulations can be found in *Supplementary 5* and *6*.

## 3. Results

### 3.1. Temporal Dynamics of ASF Transmission

**Figure 3:**
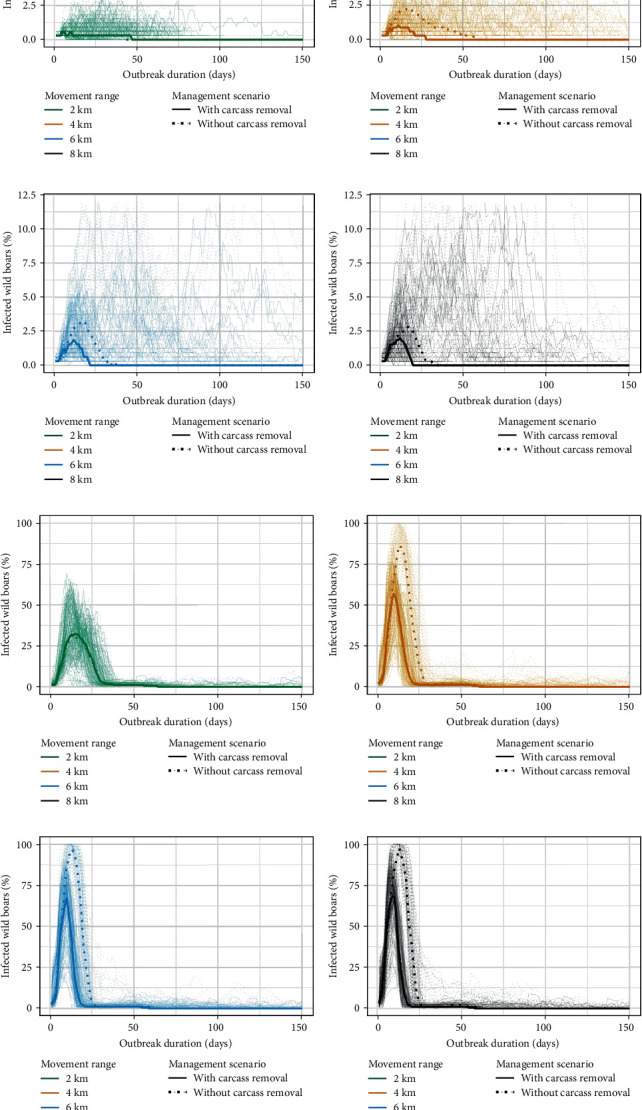
Comparison of active (solid line; with carcass removal) and passive (dotted line; without carcass removal) management scenario and wild boar dispersal capacity on the simulated ASF infection dynamics in Singapore. (a–d) Simulated temporal dynamics of infectious wild boars on mainland Singapore. (e–h) Simulated temporal dynamics of infectious wild boars on Pulau Ubin. (a–h) Median model projections for each management scenario are shown as bold lines while the trajectories for each simulation are shown as thin lines.

The infection dynamics of ASF in wild boars based on our simulations for different carcass management scenarios and dispersal capacity are shown in Figure 3 and Table 3. Based on the hypothesized disease landing points, the ASF outbreak was localized to two areas: (i) Pulau Ubin, and (ii) western part of mainland Singapore. Our model projections showed that the ASF epidemic peaked within 3 weeks of disease introduction, with the highest number of infections occurring between day 9 and 15 in Pulau Ubin, and between day 11 and 17 on mainland Singapore. Increasing the dispersal capacity of the wild boars resulted in higher number of infections, with greatest sensitivity observed when increasing the dispersal capacity of wild boars from 2 to 4 km. The infection trajectories, however, were similar at dispersal distance of 6 and 8 km, with <1% change to the proportion of infected population for both Pulau Ubin and mainland populations.

The median epidemic duration on Pulau Ubin ranged between 57 and 67 days, with >98% of the wild boar population estimated to succumb to the disease (Table 3). Carcass removal only had a marginal impact on the infection trajectory, epidemic duration, and total mortality projected on Pulau Ubin. In contrast, the outbreak on mainland Singapore was shorter and estimated to last between 20 and 57 days. Increasing the dispersal capacity of wild boars (i.e. 6 and 8 km), and simulating carcass removal and decontamination appeared to further reduce the epidemic duration by 13.5 and 4.4 days on mainland Singapore and Pulau Ubin, respectively. The overall mortality was lower on mainland Singapore compared to Pulau Ubin, with a projected mortality of 3%–7% across all dispersal distances (Table 3). The percentage of iterations where the epidemic reoccurred through infectious carcasses ranged from 18% to 75% in our simulations, highlighting the impact of carcass-mediated transmission on the persistence of ASF. Carcass removal and decontamination efforts reduced the incidence of reoccurring epidemics through infectious carcasses by 10.8% on mainland Singapore and 2.5% on Pulau Ubin (Table 3).

### 3.2. Spatial Extent of ASF Transmission

**Figure 4:**
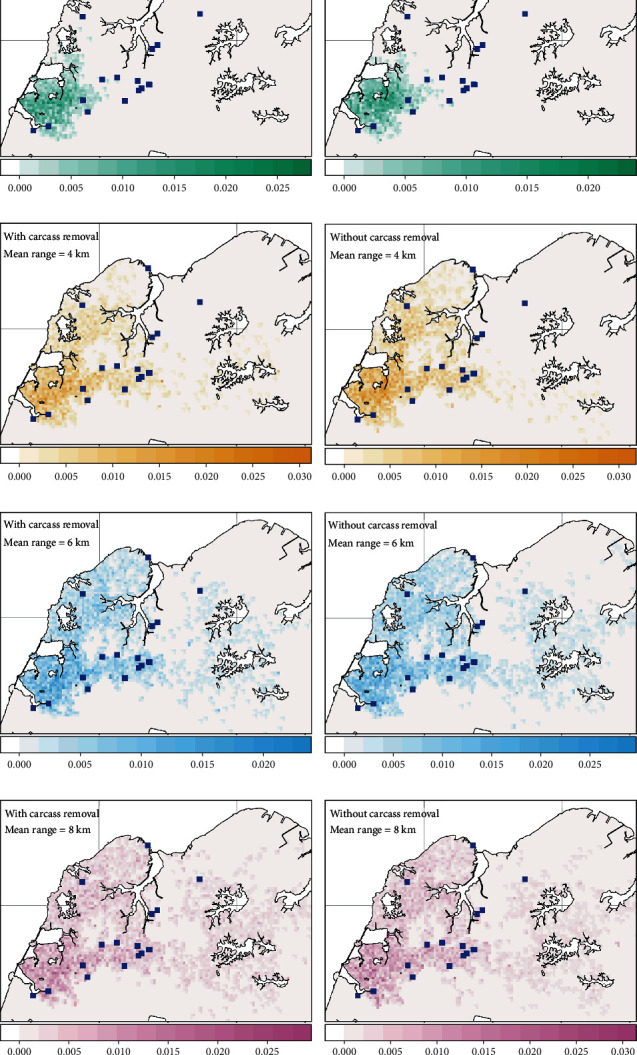
Spatial distribution of simulated wild boar mortalities on Singapore's mainland under different carcass management scenarios and dispersal capacities (2, 4, 6, and 8 km) of wild boars. (a, c, e, and g) Probability of mortality with carcass removal. (b, d, f, and h) Probability of mortality without carcass removal. (a–h) Blue squares indicate wild boar carcasses reported between February to March 2023.

**Figure 5:**
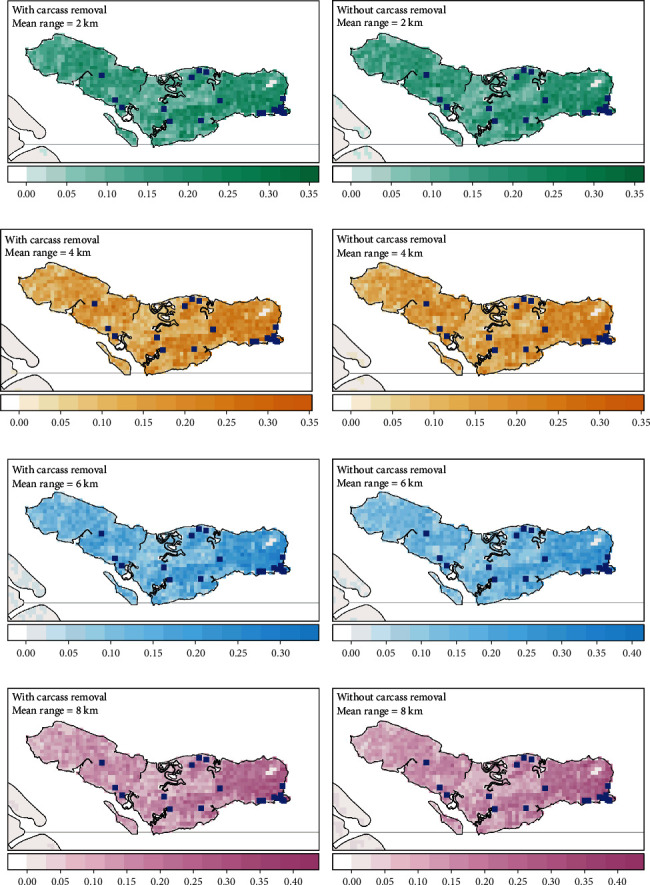
Spatial distribution of simulated wild boar mortalities on Pulau Ubin under different carcass management scenarios and dispersal capacities (2, 4, 6, and 8 km) of wild boars. (a, c, e, and g) Probability of mortality with carcass removal. (b, d, f, and h) Probability of mortality without carcass removal. (a–h) Blue squares indicate wild boar carcasses reported between February to March 2023.

**Figure 6:**
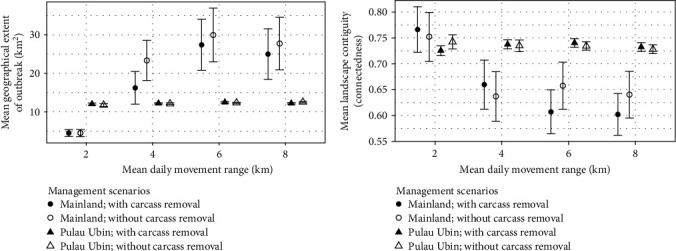
Comparison of different management scenarios (with and without carcass removal) and wild boar dispersal capacity on (a) the mean (±95% CI) maximal geographical extent of the ASF infection, and (b) the mean (±95% CI) land-use contiguity (core and dispersal habitat connectedness) in Pulau Ubin and mainland of Singapore.

Our model projections corroborate with the observed ASF mortalities reported between February and March 2023 in Singapore (Figures 4 and 5). The mean maximal geographic extent of the ASF outbreak projected on the mainland Singapore was highly variable, ranging from 5 to 30 km^2^ (Figure 6). At reduced dispersal distances (2 and 4 km), the infected land area was limited to only the western part of Singapore (Figure 4). Increasing the dispersal distances (6 and 8 km) of wild boars on mainland Singapore resulted in larger infected land area (up to 30 km^2^), where the disease was projected to spread to the CCNR (Figure 4). However, no sustained infections were observed to occur in CCNR during Singapore's outbreak. In Pulau Ubin, the disease was projected to spread from the northwestern region throughout the entire island with a mean total infected land area of 11.8–12.5 km^2^ (Figure 6). Majority of the carcasses were also projected to occur on the eastern coastline of the island, which coincided with the locations where more than 40% of the ASF-infected carcasses were reported during the specified period in early 2023 (Figure 5).

In general, the landscape contiguity for the simulated infected land was higher for Pulau Ubin than on the mainland Singapore (Figure 6), suggesting that the core and dispersal habitat patches are more connected. The landscape contiguity of the infected land area was similar between Pulau Ubin and mainland Singapore at the 2 km dispersal scenario. However, the geographic extent of the outbreak was larger in Pulau Ubin, suggesting that the differences in wild boar density might be a contributing factor to the larger infected surface area projected for Pulau Ubin. While carcass removal affects the temporal progression of the epidemic, it has little effect on the maximal geographical extent of the outbreak (Figure 6), and the spatial distribution of carcasses in Pulau Ubin (Figure 5).

## 4. Discussion

In this study, we employed an agent-based model to predict the fine-scale spatiotemporal transmission dynamics of the first reported ASF outbreak in Singapore's wild boar population. Even though the cross-border movement of wild boars was not highlighted by Singapore as one of the risk pathways in the subregional ASF cross-border risk assessment [60], the model supports our initial hypothesis that the disease was likely introduced through infected wild boars entering Singapore from affected regions [16, 61]. Using our individual-based approach, we simulated each agent's movement decisions based on their underlying land-use habitat and investigated the ASF epidemiology and disease persistence under varying carcass management actions and wild boar dispersal capacity. Overall, our model demonstrated that the ASF outbreak in Singapore was short-lived, with no additional carcass reports made at the time of writing.

The spatial extent of ASF transmission typically depends on the pathogen features (e.g., virulence and infectivity), the characteristics of the host population (e.g., density and dispersal), and the landscape connectivity [32, 62]. Landscape connectivity and habitat availability are important factors that facilitate the transboundary dispersal of wild boars and the transmission of ASF in Europe [32, 63]. In the context of Singapore, the ASF outbreak was more severe in Pulau Ubin, which has high wild boar abundance and forest cover [15]. Pulau Ubin is less urbanized and the forest patches are highly connected by open wildlife passages. This allowed the wild boars to move and interact freely regardless of their dispersal capacity, contributing to the extensive and rapid spread of ASF throughout the entire island and population. On the mainland, however, only 4% of the population was infected.The outbreak on mainland Singapore was largely localized to the western catchment owing to greater habitat heterogeneity and low wild boar density. These intrinsic attributes (low density and habitat connectedness) might have reduced the contact rate among wild boars and limited the spread of ASF on mainland Singapore, despite having larger habitat patches. Similarly in Finland or Sweden which have vast coverage of suitable habitats for wild boars, the risk for ASF transmission in these habitats was low owing to low wild boar abundance, which reduces the overall connectivity and interaction among agents [32].

Despite having a more heterogeneous landscape comprising of different interspersed habitat types on mainland Singapore, our model showed that the disease could potentially spread from the western catchment into the CCNR through dispersal corridors (e.g., riparian vegetation and open pervious surfaces) when wild boars exhibited higher dispersal activity. Other studies have shown that wild boars are able to exploit features such as green corridors, water courses, and human-built landscapes (e.g., buildings and roads) to disperse long distances or infiltrate into urban areas [30, 38, 64]. In Singapore, satellite tracking and genetic evidence have shown that wild boars are able to disperse across highly urbanized landscapes to colonize forest patches within the CCNR [15, 26].

The ASF outbreak in Singapore was estimated to peak within 3 weeks after disease introduction. This is consistent with other simulation studies in high-density populations owing to the virulence of ASFV [21, 65]. Our model assumed that infected wild boars were unlikely to disperse across large water bodies such as reservoirs or coastal barriers, which explained why the disease was largely confined within the island boundaries of Pulau Ubin. While coastal barriers may limit the dispersal of infected wild boars, we note that wild boars are capable of swimming across rivers, lakes, and out to sea [66]. Numerous studies have shown that wild boars are able to exploit riparian vegetation or waterways lined with dense vegetation as a natural corridor for dispersal [25, 38, 46]. Previously, anecdotal reports documenting wild boars swimming in the waters off Singapore's coastline [35, 67, 68] highlight their dispersal capabilities, suggesting that this could be one of their recolonization and disease introduction pathways into Singapore [15]. In this study, we posited that the ASF incursion occurred through the western coast of mainland Singapore and the northern coast of Pulau Ubin. The availability of suitable habitats such as dense forest patches and the general lack of urban development in the western catchment and Pulau Ubin could have attracted wild boars to colonize these areas as it has been shown that wild boars generally prefer to inhabit forests and forest edges [15, 28]. Overall, our model projections were able to identify the potential disease landing sites, and the estimated geographic extent of the infected area matches the reported ASF-infected/suspected carcass locations. In addition, the timing of Singapore's outbreak ensued with those reported from the region during the same period.

In the context of disease control, passive or syndromic surveillance has been considered an effective method for early detection under disease-free settings [62, 69, 70, 71]. According to our model, active carcass removal and decontamination measures were unable to control the geographic extent of the ASF outbreak, the speed of transmission, nor the overall mortality and morbidity rate among the infected wild boars. However, prompt carcass removal may help to shorten the epidemic duration and reduce the number of reoccurring infections through carcass-mediated transmission. Similarly, other studies demonstrated the potential for active control measures that included carcass removal and focal hunting or trapping of wild boars to reduce the population density and curb the spread of ASF [72]. However, some studies noted that hunting may lead to unintended consequences such as increasing the wild boars' dispersal activity and fomite transmission through contaminated hunting equipment or hunter's movement, leading to the geographical expansion of the outbreak in previously unaffected areas [32, 72, 73].

To optimize resources for ASF management, passive or syndromic surveillance can be used for early detection of the presence of ASFV in wild boars. Preemptive measures such as hunting and trapping may reduce the wild boar density to limit the ASF transmission [73]. From Singapore's experience, carcass removal and decontamination efforts increase the ability for detecting positive cases as well as reducing the risk of accidental introduction of contaminated fomites which is consistent with other studies [20, 73]. Additional to carcass removal and decontamination efforts, partitioning the population into segregated epidemiological units has also been suggested as a cost-effective mitigation strategy to control ASF [74]. Future research on the wild boar ecology (e.g., population and movement) will be useful in providing updated population estimates, dispersal rates, and distances for better model predictions on the dispersal routes and high-risk areas. For example, identifying dispersal corridors or high impact areas will allow mitigation measures such as fencing or trapping to be implemented more efficiently to limit the dispersal of wild boar and contain the spread of ASF [32].

As part of Singapore's vision to safeguard animal and human health, the Animal and Veterinary Service (AVS), a cluster of the National Parks Board (NParks), has a biosurveillance programme to strengthen the management of animal diseases at the preborder, border, and postborder levels. Especially with reports of ASF outbreaks in the region, surveillance of wild boar populations are important to detect moribund cases and wild boar carcasses, where blood, tissue, and environmental samples are sent for pathogen diagnostic and testing. Early detection and rapid diagnosis of ASF during this outbreak allowed site managers to implement swift measures including trapping and euthanasia of infected/exposed wild boars, removing carcasses, and decontamination of the environment. These control measures together with enhanced surveillance and close monitoring of suspected incidence of ASF cases provided insights into the distribution of wild boars and the ASF transmission dynamics in Singapore.

## 5. Conclusions

Using an individual-based approach, our model highlighted the importance of accounting for spatial heterogeneity, agents' interaction, and their dispersal capacity for epidemiological modeling. The epidemic duration, severity, and spatial scale of the ASF outbreak in the wild boars of Singapore depend on several factors, including the wild boar population density, carcass persistence, habitat connectedness, and dispersal barriers that limit the contact rate among wild boars and the ASF transmission. Implementing control measures such as carcass removal helps to reduce the viral load in the environment and shorten the epidemic duration, highlighting the importance of disease surveillance and early detection for the management and control of infectious diseases.

## Figures and Tables

**Table 1:** Ecological movement parameters and activity pattern assumptions applied to the model.

Parameter	Description	Value	References
Habitat types	Land use types representing core habitat.	(a) Vegetation with limited human management (with tree canopy) (b) Vegetation with limited human management (without tree canopy) (c) Vegetation with structure dominated by human management (with tree canopy) (d) Vegetation with structure dominated by human management (without tree canopy)	Thurfjell et al. [30]; Castillo-Contreras et al. [25]; Gaw et al. [24]; Halasa et al. [18]; Khoo et al. [28]; Lecis et al. [46]; Salazar et al. [31]; Ciach et al. [29]; Goicolea et al. [32]
	Land use types representing dispersal habitat.	(a) Nonvegetated pervious surfaces (b) Freshwater swamp forest (c) Freshwater marsh (d) Mangrove forest	
	Land use types representing barrier habitat.	(a) Buildings (b) Artificial impervious surfaces (c) Reservoirs	

Mean movement distance	Euclidean distance traveled per day.	2, 4, 6, and 8 km	Campbell and Long [41]; Podgórski et al. [42]; Franckowiak et al. [43]; Brogi et al. [44]; Miettinen et al. [45]

Temporal resolution	The amount of time (min) per time-step calibrated from the mean daily distances (2, 4, 6, and 8 km, respectively).	24, 13, 9, and 7 min per time-step	*Supplementary 2*

Movement decision	Habitat-dependent movement decision by agent at each time-step.	Bernoulli; *p*_move_ = 1 – *p*_habitat use_	—

Probability of core habitat use	The probability that an agent will utilise the following core habitat classes: (a) Coniferous forest (b) Deciduous forest (c) Green area	0.7	Stillfried et al. [47]
Probability of dispersal habitat use	The probability that an agent will utilise the following dispersal habitat classes: (a) Agriculture (b) House with gardens (c) Swamp (d) Trails	0.3	

**Table 2:** Model parameters, the highest posterior density interval (HPD) from the approximate Bayesian computation (sequential Monte Carlo algorithm), and the final parameter values applied to the agent-based model to simulate African swine fever transmission in Singapore's wild boar population.

Parameter	Median (95% HPD)	Value used in model	Source
*β*_I_: Transmission probability by an infected agent	0.77 (0.55, 1.0)	Bernoulli, *p*=0.8	de Carvalho Ferreira et al. [48]; Eblé et al. [50]
*β*_D_: Transmission probability through contact with an infectious carcass	0.75 (0.52, 0.98)	Bernoulli, *p*=0.8	Guinat et al. [49]; Gallardo et al. [52]
*β*_R_: Transmission probability by a carrier (recovered) agent	0.52 (0.07, 0.99)	Bernoulli, *p*=0.5	Eblé et al. [50]
*μ*_A_: Probability of death following acute infection	0.89 (0.80, 0.98)	Bernoulli, *p*=0.9	Walczak et al. [53]
*μ*_C_: Probability of death following chronic infection	—	Bernoulli, *p*=0.45	Assumed as 50% of acute infection
*γ*: Probability to recover from the disease	0.10 (0.02, 0.19)	Bernoulli, *p*=0.1	Inversely proportional to *μ*_A_
*σ*: Severity of infection	—	Bernoulli, *p*=0.9	Gallardo et al. [52]
Duration of incubation period for exposed agents	4 (3, 5.9)	Poisson, *λ* = 7 days	Guinat et al. [49]; Walczak et al. [53]; Lee et al. [54]
Duration of infectious period of carrier (recovered) agents	—	Poisson, *λ* = 24 days	Eblé et al. [50]
Duration of carcass persistence in tropics (without and with carcass removal)	—	Poisson, *λ* = 10 and 3 days	Mazur-Panasiuk et al. [55]; Nuanualsuwan et al. [56]; Lamperty et al. [15]; Leivers et al. [7]
Time to death following an acute infection	—	Poisson, *λ* = 4 days	Walczak et al. [53]
Time to death following a chronic infection	—	Poisson, *λ* = 24 days	Eblé et al. [50]
Duration of immune period following recovery from the disease	—	Poisson, *λ* = 24 days	Eblé et al. [50]
Mean distance traveled per day	—	4 km	Janeau et al. [57]

**Table 3:** The estimated outbreak duration and the proportion of mortalities at the end of the simulation are shown as median (SD) and the number of iterations with reoccurring epidemics through carcass-mediated transmission is shown as a percentage. The carcass persistence under passive and active management scenarios was assumed to be 10 and 3 days, respectively.

Area	Management	Daily dispersal capacity (km)	Outbreak duration (days)	Estimated mortality (%)	Iterations (%) where infection reoccurred
Mainland	Active	2	47.5 (24.3)	4.1 (3.8)	65
		4	28 (40.5)	4.2 (11.4)	33
		6	21 (41.7)	4 (18.9)	18
		8	20 (36.4)	3.2 (22.6)	18
	Passive	2	47 (22.4)	3.6 (3.4)	75
		4	56.5 (48.2)	6.9 (12.5)	46
		6	37 (43.8)	4.9 (21.8)	32
		8	31 (41.2)	3.6 (21.3)	24

Pulau Ubin	Active	2	63.5 (25.4)	98.8 (11.8)	23
		4	60 (32.6)	98.3 (13.5)	40
		6	58 (36.9)	99.4 (15.8)	31
		8	57 (29.1)	99.4 (12.7)	37
	Passive	2	64 (24.7)	98.8 (18.2)	26
		4	67 (35.2)	99.4 (16.5)	37
		6	63 (33.8)	99.4 (9.6)	36
		8	62 (36.3)	99.4 (9.3)	42

## Data Availability

The African Swine Fever outbreak location data in Singapore used to support the model can be found on the World Animal Health Information System (WAHIS-Event 4885; https://wahis.woah.org/#/in-event/4885/dashboard). Simulation codes for the agent-based model and Bayesian inference can be found in the Supplementary Materials.

## Supplementary Materials

Supplementary 1 Wild boar abundance estimates using density and land-use information.

Supplementary 2 Calibrating temporal resolution based on dispersal distances by simulating agent movement in a disease-free setting.

Supplementary 3 Approximate posterior distributions of the parameters and the comparison of repeated simulations against the timeline of reported carcasses.

Supplementary 4 R code to run the approximate Bayesian computation for estimating the parameters.

Supplementary 5 Source functions used during the simulation.

Supplementary 6 Example code for the agent-based model and simulation.
