# Supplementary material for: Modeling Singapore's First African Swine Fever Outbreak in Wild Boar Populations
Source: Transbound Emerg Dis. 2024 Aug 26;2024:5546893. doi: 10.1155/2024/5546893 (PMC12016949; doi:10.1155/2024/5546893)
Supplement: Supplementary 1 — Wild boar abundance estimates using density and land-use information. [file 5546893.f1.docx]

**S1. Extrapolating wild boar abundance used in the model**

We estimated Singapore’s wild boar population using wild boar density estimates derived from spatial mark-resight surveys conducted between 2017–2020 (Koh et al. 2018; Khoo et al. 2021). The wild boar density (individuals per km^2^) was extrapolated to the forest and land area coverage for each forested area which were roughly assigned to Western, Central, and Eastern regions on mainland Singapore, and Pulau Ubin. The parameters and values used for the extrapolation are presented in Table 1.

Table 1. The total land and forested area in each region used to extrapolate the wild boar abundance. The mean density and density ratio correction calculated from published data on the wild boars in Central Catchment Nature Reservoir (CCNR) (Koh et al. 2018; Khoo et al. 2021; Lamperty et al. 2023) and Pulau Ubin (Lamperty et al. 2023).

| Region | Total land area (km^2^) | Forested area (km^2^) | Mean density (ind./km^2^) | Density ratio (correction for offshore islands) | Land ratio (correction for mainland) |
| --- | --- | --- | --- | --- | --- |
| Central | 131.92 | 44.56 | 3.76 | - | 1 |
| Western | 92.181 | 44.85 | 3.76 | - | 0.71 |
| Eastern | 82.30 | 19.45 | 3.76 | - | 0.62 |
| Pulau Ubin | 10.03 | 5.77 | - | 8.74 | NA |

The mean population density calculated for Central Catchment Nature Reserve from published literature (Koh et al. 2018; Khoo et al. 2021) was 3.76 individuals per km^2^ and the total forested area in the central sub-population based on our virtual landscape was 44.56 km^2^. Assuming that the carrying capacity and population demographic of the wild boars were similar among the mainland sub-populations, we extrapolated the abundance for Western and Eastern regions using the forested:land area ratio. (Table 1).

The wild boar population trends in the Pulau Ubin, however, was markedly higher from the mainland based on the relative abundance estimates derived using single-species N-mixture models (Lamperty et al. 2023). Based on the detection-corrected relative abundance, the wild boar population in Pulau Ubin was estimated to two-times higher than key habitats (i.e., Mandai, Peirce Thomson, Bukit Timah, and MacRitchie) in CCNR combined (Table 2). As such, we calculated the corrected density area for Pulau Ubin by extrapolating using the relative forested area ratio between Pulau Ubin against other key habitats in CCNR. This is calculated as:

$$Corrected density ratio=\frac{Relative abundance ratio}{Pulau Ubin to CCNR area ratio}$$

Table 2. Land area information and detection-corrected relative abundance data for forest habitats included in Lamperty et al. (2023).

| Region | Key habitat | Area (km^2^) | Detection-corrected relative abundance | Relative abundance ratio | Corrected forested area ratio | Corrected density ratio |
| --- | --- | --- | --- | --- | --- | --- |
| Pulau Ubin |  | 10.2 | 60 | 2 | 0.23 | 8.74 |
| Central |  | 38.4 | 30 |  |  |  |
|  | Mandai | 11.8 | 9 |  |  |  |
|  | Upper Peirce | 14.7 | 4 |  |  |  |
|  | Bukit Timah | 1.7 | 11 |  |  |  |
|  | MacRitchie | 10.2 | 6 |  |  |  |

**References**

Khoo, M. D. Y. and others 2021. Persistence of a locally endangered mouse-deer amidst the re-emergence of two larger ungulates in small urban rainforest fragments. Global Ecology and Conservation **30:** e01807.

Koh, J. J. M., E. L. Webb, and L. K.-P. Leung. 2018. Using a spatial mark-resight model to estimate the parameters of a wild pig (*Sus scrofa*) population in Singapore. Raffles Bulletin of Zoology **66:** 494–505.

Lamperty, T. and others 2023. Rewilding in Southeast Asia: Singapore as a case study. Conservation Science and Practice **5:** e12899.
