# Supplementary material for: Modeling Singapore's First African Swine Fever Outbreak in Wild Boar Populations
Source: Transbound Emerg Dis. 2024 Aug 26;2024:5546893. doi: 10.1155/2024/5546893 (PMC12016949; doi:10.1155/2024/5546893)
Supplement: Supplementary 2 — Calibrating temporal resolution based on dispersal distances by simulating agent movement in a disease-free setting. [file 5546893.f2.pdf]

S6. Calibrating temporal resolution through movement simulation

Research on the movement ecology of wild boars is Singapore is limited to one study for one wild boar individual (Koh et al. 2018) and the trajectory and movement data is lacking owing to the challenges associated with collaring studies (i.e., malfunction, collar retention, cost). To overcome this data gap, we seek to simulate the movement of wild boars and estimate the temporal resolution for the agent-based model using movement parameters (e.g., daily mean movement distance) from published sources (Table 1).

Table 1. Mean daily distance travelled by wild boars reported from other studies.

**Mean distance (km/day)**	**Standard error (SE)**	**References**
4.66	1.08	Campbell and Long (2010)
9.85	1.62	Podgórski et al. (2013)
3.71	0.09	Franckowiak et al. (2018)
2.89	0.12	Brogi et al. (2023)
5.43	0.37	Miettinen et al. (2023)

The temporal resolution of the simulation was determined in a disease-free setting. At the start of each iteration, each agent will be randomly assigned to a Core habitat cell type. A Bernoulli process was used to determine the agent's movement decision based on its underlying habitat. For example, when the agent inhabits a Dispersal habitat type (i.e. open and semi-natural areas), they exhibit a greater tendency to continue moving until they occupy a Core habitat (e.g., forests) where they will spend more time foraging, resting, or seeking refuge. The agent will be tracked over 3000 time-steps and the positions are recorded to calculate the Euclidean distance covered between time-steps (Figure 1). The simulation was conducted for 100 agents and the cumulative Euclidean distance covered by each agent was analyzed using linear regression (Figure 2) to estimate the timesteps required to achieve the target mean distance of 2, 4, 6, and 8 km. The estimated time-steps are presented in Table 2.

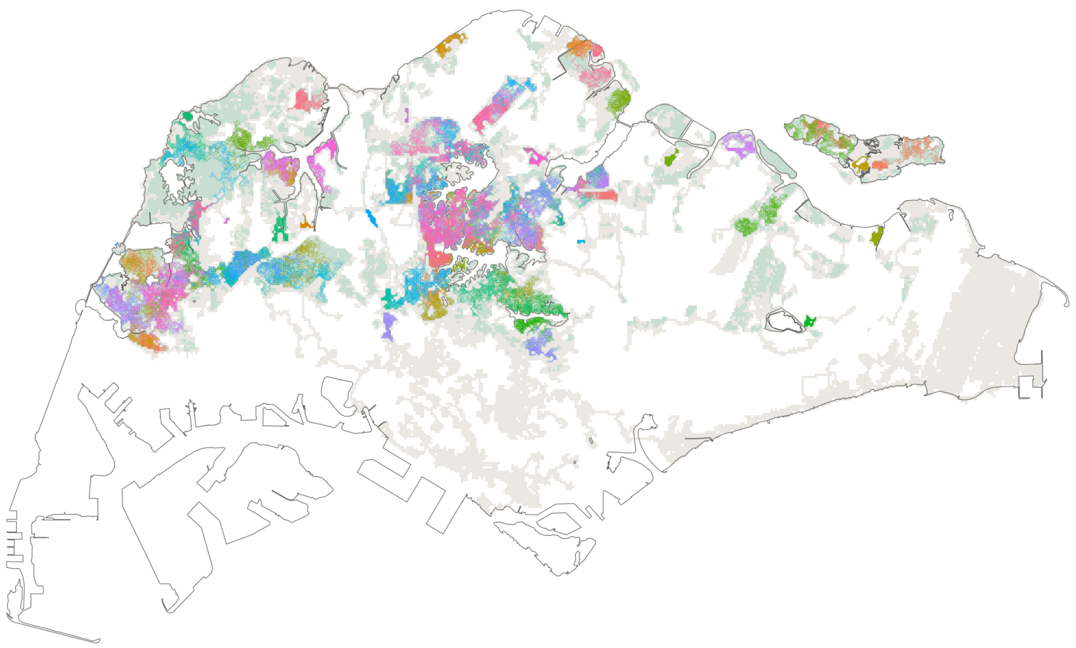

Figure 1. Map of Singapore used for the movement simulation in a disease-free setting. Teal areas represent Core habitat while grey areas represent Dispersal habitat. The movement trajectory of each agent is indicated as coloured paths.

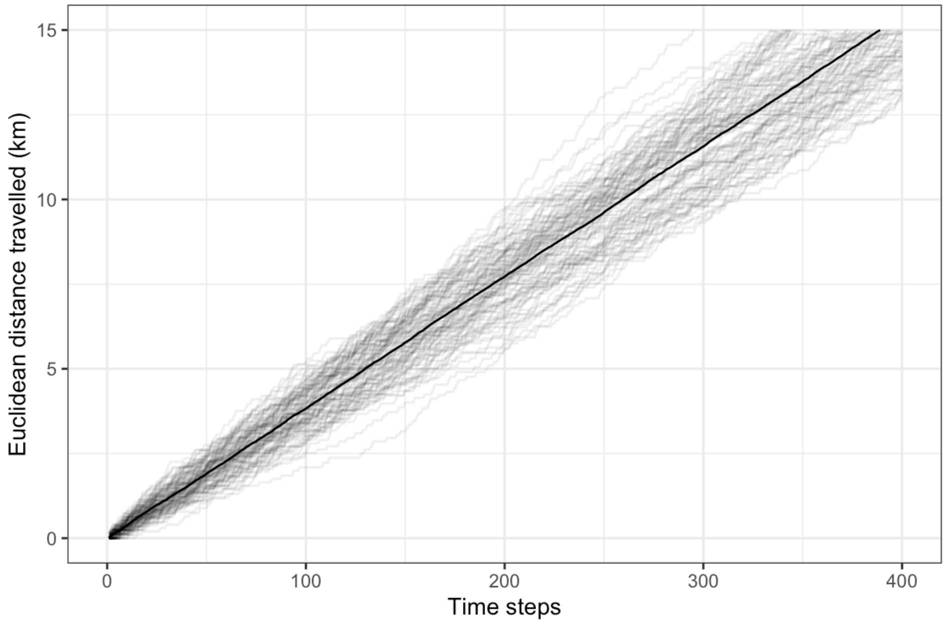

Figure 2. Cumulative distance travelled by each agent at each time-step are represented by the grey lines. The black line represents the mean distance travelled per time-step across all 100 individuals.

Table 2. Estimated time-step predicted from the linear regression.

**Mean daily distance (km)**	**Time-step**	**Standard error**	**Temporal resolution (time per time-step)**
2	60	0.26	24 min
4	110	0.25	13 min
6	165	0.25	9 min
8	215	0.24	7 min

References:

Brogi, R., M. Apollonio, S. Grignolio, A. Cossu, S. Luccarini, and F. Brivio. 2023. Behavioural responses to temporal variations of human presence: Insights from an urban adapter. Journal of Zoology **321:** 215–224.

Campbell, T. A., and D. B. Long. 2010. Activity patterns of wild boars (Sus scrofa) in southern Texas. The Southwestern Naturalist **55:** 564–567.

Franckowiak, G. A., Z. Torres-Poché, and R. M. Poché. 2018. Activity patterns by feral hogs in the Texas panhandle. The American Midland Naturalist **180:** 233–245.

Koh, J. J. M., E. L. Webb, and L. K.-P. Leung. 2018. Using a spatial mark-resight model to estimate the parameters of a wild pig (*Sus scrofa*) population in Singapore. Raffles Bulletin of Zoology **66:** 494–505.

Miettinen, E. and others 2023. Home ranges and movement patterns of wild boars (Sus scrofa) at the northern edge of the species' distribution range. Mammal Research **68:** 611–623.

Podgórski, T. and others 2013. Spatiotemporal behavioral plasticity of wild boar (Sus scrofa) under contrasting conditions of human pressure: primeval forest and metropolitan area. Journal of Mammalogy **94:** 109–119.
