# Supplementary material for: Modeling Singapore's First African Swine Fever Outbreak in Wild Boar Populations
Source: Transbound Emerg Dis. 2024 Aug 26;2024:5546893. doi: 10.1155/2024/5546893 (PMC12016949; doi:10.1155/2024/5546893)
Supplement: Supplementary 3 — Approximate posterior distributions of the parameters and the comparison of repeated simulations against the timeline of reported carcasses. [file 5546893.f3.pdf]

S4. Approximate posterior distributions of model parameters

The approximate posterior distributions of the transmission rates (infected, carriers, and carcass), recovery rate, incubation period, and the mortality rate are presented in Figure 1. These distributions were estimated using the Sequential Monte Carlo algorithm which was used to sample combinations of the model parameters (i.e., particles) based on a distance function (Le et al. 2023).

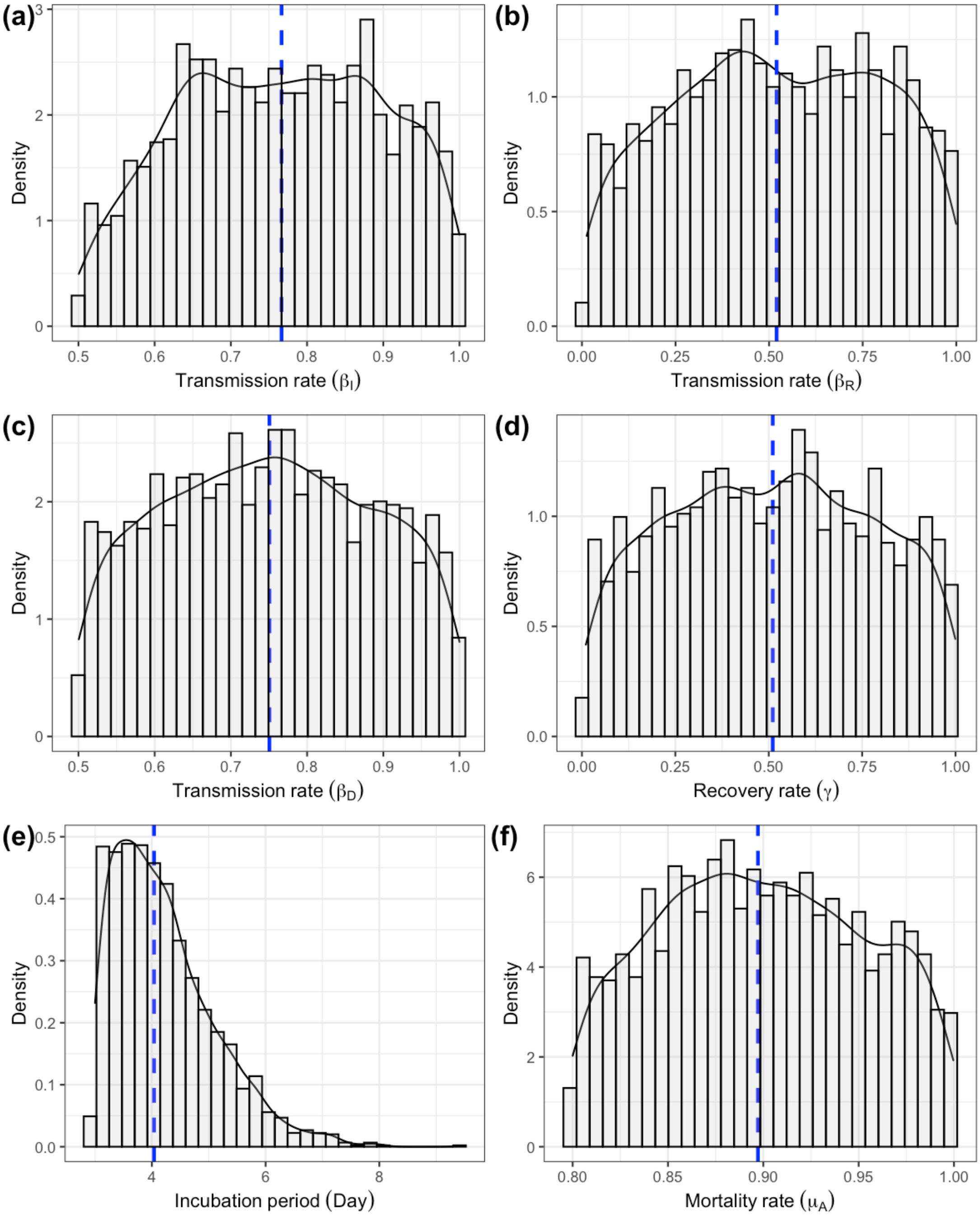

Figure 1. Approximate posterior distributions of the model parameters (i.e., transmission, recovery, incubation period, and mortality).

The distance function calculates the difference between 1) observed versus simulated days to reach epidemic peak, observed vs simulated peak wild boar deaths, and 3) the sum of the epidemic curve residuals (i.e., difference in epidemic duration). The thresholds used for the distance function are ±4 days difference to reach epidemic peak, ±4 deaths at the epidemic peak, and ±40 days for the differences in epidemic duration. The epidemic model used in the Approximate Bayesian Computation was modified to include carcass-mediated and covalescent transmission (Gervasi and Guberti 2021).

The epidemic curve presented in the main paper (main paper: Figure 3) was simulated by taking the point-wise estimate (i.e., median) of the approximate posterior distributions. To check that the estimated model parameters converge to the true underlying density, we re-simulated 1000 trajectories and compared it with the empirical data (i.e., reported deaths). From the re-simulations, 39.5% of the iterations met the tolerance criteria to reach peak epidemic within ±1 day, 21.5% met the tolerance criteria to predict the peak number of deaths within ±1 case, and 100% met the tolerance for epidemic duration with residual sums withint ±40 days.

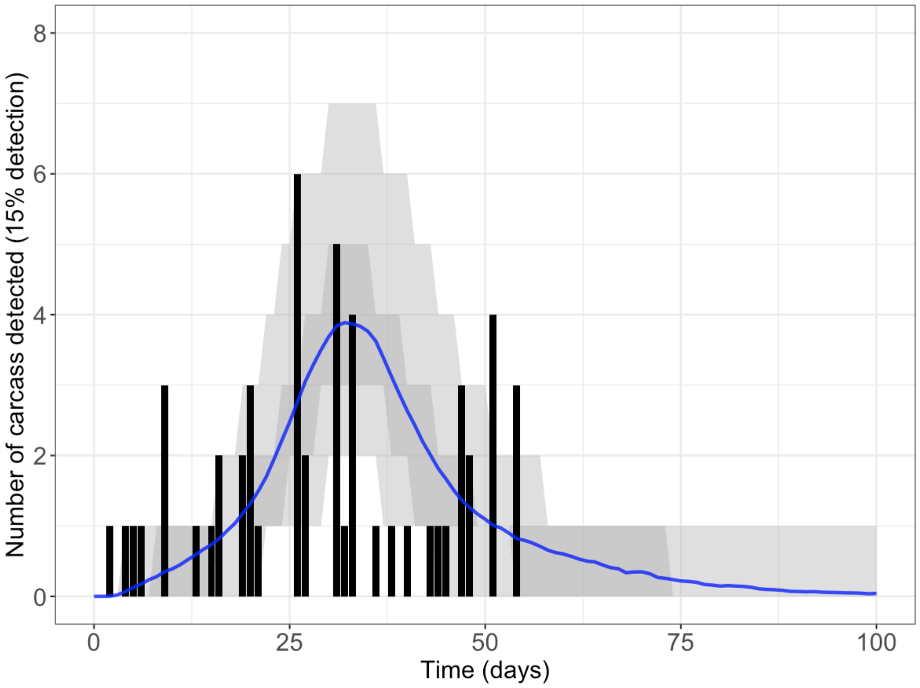

Figure 2. Bar plot showing the number of reported dead wild boars as a function of the number of days since the disease incurred (black bars). The predicted mean number of dead wild boars based on 1000 simulated trajectories sampled from the posterior is represented by the blue line. The dark and light grey shaded areas represent the 50% and 95% quantiles around the mean.

References

Gervasi, V., and V. Guberti. 2021. African swine fever endemic persistence in wild boar populations: key mechanisms explored through modelling. Transboundary and Emerging Diseases **68:** 2812–2825.

Le, V. P. and others 2023. Estimation of a within-herd transmission rate for African swine fever in Vietnam. Animals **13:** 571.
