# Supplementary material for: Modeling Singapore's First African Swine Fever Outbreak in Wild Boar Populations
Source: Transbound Emerg Dis. 2024 Aug 26;2024:5546893. doi: 10.1155/2024/5546893 (PMC12016949; doi:10.1155/2024/5546893)

Approximate Bayesian Computation supplementary code

Rayson Lim

2023-09-02

Approximate Bayesian Computation

This file documents the procedures and R script to run the Approximate Bayesian Computation using the Sequential Monte Carlo algorithm. The principle is to iteratively and sequentially sample from a prior distribution and at each step, weight the particles using a distance function, resample, and mutate until a posterior distribution approximating to the true density is obtained.

Empirical data (carcass reports)

Observed infected/suspected carcass reports (assumed epidemic started 2 weeks prior to report from the index case)

```
D<- c(0, 0, 1, 0, 1, 1, 1, 0, 0, 3, 0, 0, 0, 1, 0, 1, 2, 0, 0, 2, 3, 1, 0, 0, 0, 0, 6, 2, 0, 0, 0, 5, 1
time<- c(1:length(D))
df.obs<- data.frame(time, D)
```

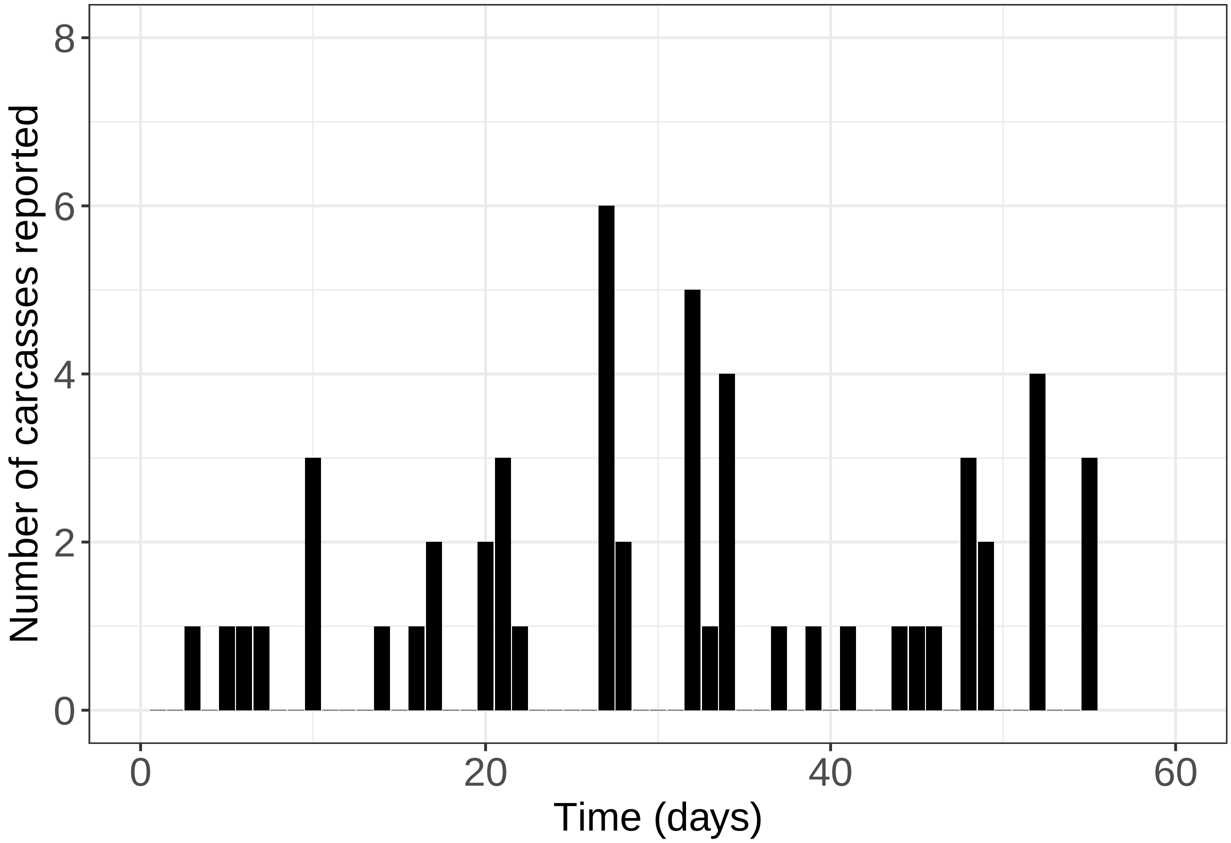

Compartmentalized model (SEIRD)

Custom functions to implement the SIR model to include the effect of carcass-mediated and covalescent (i.e. carrier) tranmissions. The incubation and latent period was assumed to be one week.

```
## Epidemic model modified to include carcass-mediated and covalescent transmission
#One-step model
SEIRDmodel<- function(x, params){ #function to calculate one step of stochastic SIR

  t  <- x[1] #local variable for time

  S  <- x[2] #local variable for susceptibles

  E1 <- x[3]
  E2 <- x[4]
  E3 <- x[5]
  E4 <- x[6]
  E5 <- x[7]
  E6 <- x[8]
  E7 <- x[9]

  I1  <- x[10]
  I2  <- x[11]
  I3  <- x[12]
  I4  <- x[13]
```

```
I5  <- x[14]
I6  <- x[15]
I7  <- x[16]

R  <- x[17]

D  <- x[18]

Dc <- x[19]

N = sum(x[2:18])

beta1 = params[1]
beta2 = params[2]
beta3 = params[3]
sigma = params[4]*7
gamma = params[5]*6
delta = params[6]
mu = params[7]
lambda = params[8]
alpha = params[9]


rates <- c(beta1*S*(I1+I2+I3+I4+I5+I6+I7)/N, #infected by infectious agents
           beta2*S*R/N, #infected by carrier agents
           beta3*S*D/N, #infected by dead carcass
           alpha*R, #recovered agents past immune period

           sigma*E1,
           sigma*E2,
           sigma*E3,
           sigma*E4,
           sigma*E5,
           sigma*E6,
           sigma*E7,

           gamma*I1,
           gamma*I2,
           gamma*I3,
           gamma*I4,
           gamma*I5,
           gamma*I6,

           delta*I7,

           mu*I7,

           lambda*D
)


changes <- matrix(
  c(-1,1,0,0,0,0,0,0,0,0,0,0,0,0,0,0,0,0, #beta1*S*I
```

```
      -1,1,0,0,0,0,0,0,0,0,0,0,0,0,0,0,0,0, #beta2*S*R
      -1,1,0,0,0,0,0,0,0,0,0,0,0,0,0,0,0,0, #beta2*S*D
      1,0,0,0,0,0,0,0,0,0,0,0,0,0,0,-1,0,0, #alpha*R (full recovery)

      0,-1,1,0,0,0,0,0,0,0,0,0,0,0,0,0,0,0, #sigma*E1
      0,0,-1,1,0,0,0,0,0,0,0,0,0,0,0,0,0,0, #sigma*E2
      0,0,0,-1,1,0,0,0,0,0,0,0,0,0,0,0,0,0, #sigma*E3
      0,0,0,0,-1,1,0,0,0,0,0,0,0,0,0,0,0,0, #sigma*E4
      0,0,0,0,0,-1,1,0,0,0,0,0,0,0,0,0,0,0, #sigma*E5
      0,0,0,0,0,0,-1,1,0,0,0,0,0,0,0,0,0,0, #sigma*E6
      0,0,0,0,0,0,0,-1,1,0,0,0,0,0,0,0,0,0, #sigma*E7

      0,0,0,0,0,0,0,0,-1,1,0,0,0,0,0,0,0,0, #gamma*I1
      0,0,0,0,0,0,0,0,0,-1,1,0,0,0,0,0,0,0, #gamma*I2
      0,0,0,0,0,0,0,0,0,0,-1,1,0,0,0,0,0,0, #gamma*I3
      0,0,0,0,0,0,0,0,0,0,0,-1,1,0,0,0,0,0, #gamma*I4
      0,0,0,0,0,0,0,0,0,0,0,0,-1,1,0,0,0,0, #gamma*I5
      0,0,0,0,0,0,0,0,0,0,0,0,0,-1,1,0,0,0, #gamma*I6

      0,0,0,0,0,0,0,0,0,0,0,0,0,0,-1,1,0,0, #delta*I7

      0,0,0,0,0,0,0,0,0,0,0,0,0,0,-1,0,1,0, #mu*I7

      0,0,0,0,0,0,0,0,0,0,0,0,0,0,0,0,-1,1  #lambda*D
    ), ncol = 18, byrow = TRUE)


  tau <- rexp(n=1,rate=sum(rates)) # exponential waiting time
  U <- runif(1) #uniform random deviate
  m <- min(which(cumsum(rates)>=U*sum(rates)))
  x <- x[2:19] + changes[m,]
  t <- t + tau
  return(out <- c(t, x))
}

#Simulating the whole epidemic
SEIRD.stoc.model <- function (xstart, params, tfinal) { #function to simulate stochastic SIR
  output <- array(dim=c(1,19)) #set up array to store results
  colnames(output) <- c("time",
                        "S",
                        "E1","E2","E3","E4","E5","E6","E7",
                        "I1","I2","I3","I4","I5","I6","I7",
                        "R",
                        "D",
                        "Dc") #name variables
  output[1,] <- xstart #first record of output is initial condition
  k=1
  while (as.logical(output[k,1]<tfinal)){
    out.t <- SEIRDmodel(output[k,],params)
    output <- rbind(output,out.t)
    checkEnd <- sum(out.t[3:18]) <= 0
    if(checkEnd){break}
    k=k+1
```

```
  }
  rval <- as.data.frame(output, row.names = F)
  return(rval) #return output
}

#Wrapper function to summarize the output for comparison (number of deaths)
myModel <- function(par){
  init.state <- c(time=0,S=308,E1=0,E2=0,E3=0,E4=0,E5=0,E6=0,E7=0,I1=1,I2=0,I3=0,I4=0,I5=0,I6=0,I7=0,R=[illegible]
  params <- par
  simu <- SEIRD.stoc.model(init.state,params,90)
  simu$D <- ceiling(simu[,c(18)]/15) ## Need to correct for detection of carcass (set at 15%)
  return(simu)
}
```

Summary statistics for the ABC-SMC

Define the set of summary statistics for the ABC-SMC. 1. Peak infection 2. Days to epidemic peak 3. Epidemic curve residuals

```
# maximum incidence along the whole trajectory
ssMax <- function(outGill) {
  return(max(outGill$D, na.rm = T))
}

# timing of the epidemic peak
ssMaxTime <- function(outGill) {
  return(round(min(outGill[which(outGill$D == max(outGill$D)), ]$time, na.rm = T)))
}

# epidemic curve residuals
ssResids <- function(outGill) {

  t.id<-vector()
  for(i in 1:length(df.obs$time)){
    t.id[i]<-which.min(abs(outGill$time - df.obs$time[i]))
  }
  return(sum(abs(outGill$I[t.id] - df.obs$D)))
}
```

Priors and target (distance threshold)

```
#define priors
myPriors <- list('beta1' = c("unif",0.5,1), ##Infected
                 'beta2' = c("unif",0.01,1), ##Carrier
                 'beta3' = c("unif",0.5,1), ##Dead
                 'sigma' = c("unif",1/14,1/3), ##Incubation period
                 'gamma' = c("unif",1/20,1/5), ##Infectious period
                 'delta' = c("unif",0.01,0.2), ## Recovery rate
                 'mu' = c("unif",0.8,1), ## Death rate
                 'lambda' = c("unif",0.01,1), ##Decomposition (inverse to obtain decomposition period)
```

```
                 'alpha' = c("unif",0.01,1)) ##Full recovery to susceptible


#define targets
myTarget <- c(ssMax(df.obs), ssMaxTime(df.obs), 0)
```

ABC-SMC routine

Preparing the matrices and specifications for the ABC step.

```
epsilon.step = 1
epsilon.steps = 5 #Increase for better accuracy (original run = 20 steps)
n.particles = 20 #Increase for better accuracy (original run = 2000 particles)


#tolerances
#In the first step, particles were accepted if their distance from the observed data was within predefi
epsilon.t <- matrix(NA, nrow=epsilon.steps, ncol=3)

## +/- 4 days to capture epidemic peak, with +/- 4 mortality at the peak and epidemic residual sum of <
epsilon.t[1,] <- c(4,4,40)

#proposal function to sample randomly from the prior range uniformly
proposal_func<-function(priors){
  sapply(priors,FUN = function(x) switch(x[1],
                                          unif=runif(n=1,min=as.numeric(x[2]),max=as.numeric(x[3])))
  )}

proposal_func(myPriors)
```

```
##     beta1     beta2     beta3     sigma     gamma     delta        mu    lambda
## 0.7119985 0.9911394 0.9057492 0.3093289 0.1929349 0.0694095 0.8902189 0.3339191
##     alpha
## 0.9975504
```

```
#prior probability function to ensure that the newly sampled particles falls within the prior range
prior_pr_func<-function(theta.proposed, priors){

  ret<-rep(NA, length(priors))

  for(p in 1:length(priors)){

    if(priors[[p]][1]=='unif'){
      ret[p]<-ifelse(theta.proposed[p]>=as.numeric(priors[[p]][2]) &
                       theta.proposed[p]<=as.numeric(priors[[p]][3]),1,0)
    }
    #if other prior options can go here
  }
  return(ret)
}

prior_pr_func(proposal_func(myPriors), myPriors)
```

```
## [1] 1 1 1 1 1 1 1 1 1
```

```
#matrix to store weights per particle per SMC step
weights <- matrix(1/n.particles,epsilon.steps,n.particles)

#arrays to store inferred parameter sets per particle per step
theta.a<-array(NA,dim=c(epsilon.steps,n.particles,length(myPriors)))
#matrices to store weighted estimates of theta mean and variance per particle per step
tau.mu.a<-matrix(NA,epsilon.steps,length(myPriors))
tau.var.a<-matrix(NA,epsilon.steps,length(myPriors))

#matrixes to store distances - per tolerance metric, per particle, per step
d1  <- matrix(NA,epsilon.steps,n.particles) ## Days to reach mortality peak
d2  <- matrix(NA,epsilon.steps,n.particles) ## Peak mortality size
d3  <- matrix(NA,epsilon.steps,n.particles) ## Epidemic curve residuals (by day)


#distance function - from summary statistic(s)
dfunc<-function(x,y){abs(x-y)} #euclidean distance, separately for each tolerance


#matrices to store outputs per timestep - for subsequent posterior sampling
x.a<-list(vector("list", n.particles))

x.a<-vector("list", epsilon.steps)
for(i in 1:epsilon.steps){
  x.a[[i]]<-vector("list", n.particles)
}


#matrix for calculating acceptance %s per step
j.it.s  <- matrix(NA,epsilon.steps,n.particles)
```

ABC-SMC steps

The set of code runs the Sequential Monte Carlo algorithm based on the number of pre-defined steps and number of particles. Note: this loop takes several hours/days to run (depending on the particle and step size). To test the code, run less epsilon.steps and reduce number of particles (n.particles). For the actual run, it will be important to run until there is diminished gain.

```
library(progress)

for(s in 1:epsilon.steps){

  ### Set up progress bar to monitor the ABC-SMC
  pb_s <- progress_bar$new(format = "(:spin) [:bar] :percent [Elapsed time: :elapsedfull || Estimated ti
                         total = n.particles,
                         complete = "=",   # Completion bar character
                         incomplete = "-", # Incomplete bar character
                         current = ">",    # Current bar character
                         clear = FALSE,    # If TRUE, clears the bar when finish
                         width = 100)      # Width of the progress bar
```

```
pb_s$message(paste("Epsilon step:", s, "of", epsilon.steps))

if(s>1){

  #set tolerance of this step based on percentile of distances[t-1]
  epsilon.t[s,] <- c(as.numeric(quantile(d1[s-1,],.75)),
                     as.numeric(quantile(d2[s-1,],.75)),
                     as.numeric(quantile(d3[s-1,],.75)))


  for(i in 1:length(myPriors)){

    #weighted empirical mean of theta[t-1]s - log scale because ratios
    tau.mu.a[s,i]<- sum(theta.a[s-1,,i] * weights[s-1,])

    #weighted empirical variance of theta[t-1]s - log scale because ratios
    tau.var.a[s,i] <- sum((theta.a[s-1,,i] - tau.mu.a[s,i])^2 * weights[s-1,])
  }
}

for(j in 1:n.particles){
  ## ticks for progress bar
  pb_s$tick()

  j.it<-0

  d1[s,j] <- epsilon.t[s,1] + 1 #initiate with distance > epsilon.t[t]
  d2[s,j] <- epsilon.t[s,2] + 1
  d3[s,j] <- epsilon.t[s,3] + 1


  while((d1[s,j] > epsilon.t[s,1]) | (d2[s,j] > epsilon.t[s,2])
        | (d3[s,j] > epsilon.t[s,3])) {

    j.it = j.it + 1

    if(s==1){
      #draw from the prior for the first step
      theta.proposed <- proposal_func(myPriors)
    }

    if(s>1){
      #take 1 empirical weighted sample of theta.star from the theta[t-1]s
      sample.particle <- sample(n.particles, 1, prob = weights[s-1,])

      #propose based on the sampled particle
      theta.star <- theta.a[s-1,sample.particle,]

      #perturb/mutate around the proposed particle (kernel from Beaumont 2009)
      for(i in 1:length(myPriors)){

        #if uniform priors, if outside the priors resample
        theta.proposed[i]<-as.numeric(myPriors[[i]][2])-1
```

```
      while((theta.proposed[i]<as.numeric(myPriors[[i]][2])) |
            (theta.proposed[i]>as.numeric(myPriors[[i]][3]))){
        theta.proposed[i]<-rnorm(1, theta.star[i], sqrt(2*tau.var.a[s,i]))
      }

    }

  }


  #forwards process (the model) - assumes detection rate of carcass at 10%
  x <- myModel(theta.proposed)

  #calculate distance stats for this particle
  d1[s,j] <- dfunc(ssMax(x), myTarget[1]) # max death count
  d2[s,j] <- dfunc(ssMaxTime(x), myTarget[2]) # time of max death count
  d3[s,j] <- dfunc(ssResids(x), myTarget[3])

  #print(paste(j, " of ", n.particles, "; ", s, " of ", epsilon.steps ))

}

#once tolerance met, store the particle components
theta.a[s,j,]<-as.numeric(theta.proposed)

#store simulated data for posterior sampling
x.a[[s]][[j]]<-x

#store the iteration counter for calculating acceptance rates
j.it.s[s,j] <- j.it

if(s==1){}#weights remain unchanged, i.e. all = 1/N

if(s>1){

  #numerator = prior probability of theta.proposed
  weight.j.numerator<-prod(prior_pr_func(theta.proposed, myPriors))

  #denominator = weighted sum of likelihoods from the pertubation kernel
  tmp<-0
  for(i in 1:length(myPriors)){

    tmp<-tmp +
      dnorm(theta.proposed[i],theta.a[s-1,,i],sqrt(2*tau.var.a[s,i]),log=T)

  }

  weight.j.denom<- sum(weights[s-1,]*exp(tmp))

  weights[s,j] <- weight.j.numerator/weight.j.denom

}
```

```
  } #close for j n.particles

  #normalise weights to sum to 1
  weights[s,] <- weights[s,]/sum(weights[s,])

}
```

Collating outputs (posterior distributions)

This section collates the approximate posterior distributions for all the model parameters. The summary will be displayed as a table while the density distribution will be plotted. Refer to Supplementary Information (S4) for the posterior distribution plots.

```
# posterior output for each parameter
out.post <- as.data.frame(theta.a[s,,])


#calculated output R0
R0.s<-matrix(NA,n.particles,epsilon.steps)
for(s in 1:epsilon.steps){
  R0.s[,s]<-theta.a[s,,1]/theta.a[s,,5] ## Compare beta1 and gamma
}
out.post <- cbind(out.post, R0.s[,s])

#rename the posterior output for the parameters
names(out.post) <- c('beta1', 'beta2', 'beta3', 'sigma','gamma', 'delta', 'mu', 'lambda', 'alpha', 'R0'

#calculate the median and 95% credible interval for the posterior distribution for the parameter estima
library(HDInterval)
#summary stats of marginal posteriors
ro.ss <- c('R0',round(c(median(out.post$R0),hdi(out.post$R0, credMass = .95)),1), 'Basic reproduction nu
b1.ss <- c('beta1',round(c(median(out.post$beta1),hdi(out.post$beta1, credMass = .95)),1), 'Transmission
b2.ss <- c('beta2',round(c(median(out.post$beta2),hdi(out.post$beta2, credMass = .95)),1), 'Transmission
b3.ss <- c('beta3',round(c(median(out.post$beta3),hdi(out.post$beta3, credMass = .95)),1), 'Transmission
a.ss <- c('alpha',round(c(median(out.post$alpha),hdi(out.post$alpha, credMass = .95)),1), 'Probability o
s.ss <- c('1/sigma',round(c(median(1/out.post$sigma),hdi(1/out.post$sigma, credMass = .95)),1), 'Incubat
g.ss <- c('1/gamma', round(c(median(1/out.post$gamma),hdi(1/out.post$gamma, credMass = .95)),1), 'Infect
m.ss <- c('mu',round(c(median(out.post$mu),hdi(out.post$mu, credMass = .95)),1), 'Probability of death')
l.ss <- c('lambda',round(c(median(out.post$lambda),hdi(out.post$lambda, credMass = .95)),1), 'Decomposit
d.ss <- c('delta',round(c(median(out.post$delta),hdi(out.post$delta, credMass = .95)),1), 'Transition fr

out.df<- data.frame(rbind(ro.ss, b1.ss, b2.ss, b3.ss, a.ss, s.ss, g.ss, m.ss, l.ss, d.ss))
names(out.df)<- c("parameter","value","lower","upper",'description')
print(out.df)

library(ggplot2)
p1<- ggplot(out.post, aes(x=beta1)) +
  geom_density() +
  geom_vline(aes(xintercept=median(beta1)),color='blue', linetype='dashed', size=1) +
  ylab("Density") +
  xlab(expression(Transmission~rate~(beta[I])))+
  theme_bw() +
```

```
  geom_histogram(aes(y=..density..), alpha=0.2, colour='black', fill='grey')

p2<-  ggplot(out.post, aes(x=beta2)) +
  geom_density() +
  geom_vline(aes(xintercept=median(beta2)),color='blue', linetype='dashed', size=1) +
  ylab("Density") +
  xlab(expression(Transmission~rate~(beta[R])))+
  theme_bw() +
  geom_histogram(aes(y=..density..), alpha=0.2, colour='black', fill='grey')


p3<-  ggplot(out.post, aes(x=beta3)) +
  geom_density() +
  geom_vline(aes(xintercept=median(beta3)),color='blue', linetype='dashed', size=1) +
  ylab("Density") +
  xlab(expression(Transmission~rate~(beta[D])))+
  theme_bw() +
  geom_histogram(aes(y=..density..), alpha=0.2, colour='black', fill='grey')


p4<-  ggplot(out.post, aes(x=alpha)) +
  geom_density() +
  geom_vline(aes(xintercept=median(alpha)),color='blue', linetype='dashed', size=1) +
  ylab("Density") +
  xlab(expression(Recovery~rate~(gamma)))+
  theme_bw() +
  geom_histogram(aes(y=..density..), alpha=0.2, colour='black', fill='grey')


p5<-  ggplot(out.post, aes(x=1/sigma)) +
  geom_density() +
  geom_vline(aes(xintercept=median(1/sigma)),color='blue', linetype='dashed', size=1) +
  ylab("Density") +
  xlab(expression(Incubation~period~(Day)))+
  theme_bw() +
  geom_histogram(aes(y=..density..), alpha=0.2, colour='black', fill='grey')


p6<-  ggplot(out.post, aes(x=mu)) +
  geom_density() +
  geom_vline(aes(xintercept=median(mu)),color='blue', linetype='dashed', size=1) +
  ylab("Density") +
  xlab(expression(Mortality~rate~(mu[A])))+
  theme_bw() +
  geom_histogram(aes(y=..density..), alpha=0.2, colour='black', fill='grey')

library(cowplot)

### Left-Right arrangement
pall<- ggdraw() +
  draw_plot(p1, x = 0, y = 2/3, width = 1/2, height = 1/3 )+
  draw_plot(p2, x = 1/2, y = 2/3 , width = 1/2, height = 1/3) +
  draw_plot(p3, x = 0, y = 1/3 , width = 1/2, height = 1/3) +
```

```
draw_plot(p4, x = 1/2, y = 1/3 , width = 1/2, height = 1/3) +
draw_plot(p5, x = 0, y = 0 , width = 1/2, height = 1/3) +
draw_plot(p6, x = 1/2, y = 0 , width = 1/2, height = 1/3) +
draw_plot_label(label = c("(a)","(b)","(c)","(d)","(e)","(f)"), x = c(0, 1/2, 0, 1/2, 0, 1/2)-0.018, 
```

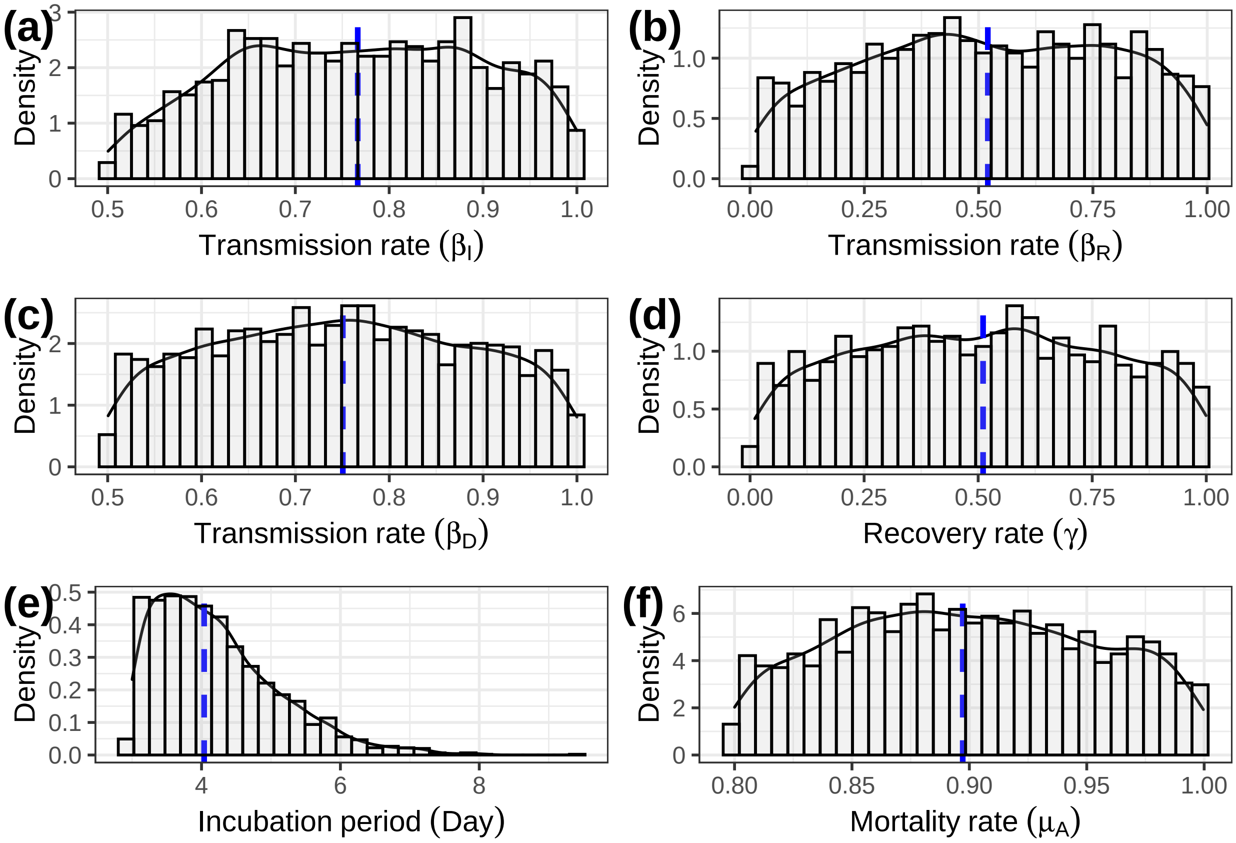

Supplement: Supplementary 4 — R code to run the approximate Bayesian computation for estimating the parameters. [file 5546893.f4.pdf]
