# Supplementary material for: Modeling Singapore's First African Swine Fever Outbreak in Wild Boar Populations
Source: Transbound Emerg Dis. 2024 Aug 26;2024:5546893. doi: 10.1155/2024/5546893 (PMC12016949; doi:10.1155/2024/5546893)
Supplement: Supplementary 5 — Source functions used during the simulation. [file 5546893.f5.pdf]

Source functions for ABM

Rayson Lim

2023-07-31

Source functions for agent-based model (ASF transmission in wild boars)

This file documents the source functions used for the agent-based simulation of the ASF transmission in the wild boars of Singapore. The functions are used to specify the movement, the transmission, incubation period, latency of the infection, recovery, deaths, and decomposition of the agents. There are also functions used to track the infection/recovery history of each agent.

```
## Function to determine neighbours (Moore's neighbourhood) in the virtual background. Need to specify
get_neighbors <- function(coordinates, raxial) {

  coordinates<- as.vector(as.matrix(coordinates))## ensure the coordinates are in the vector format

  target_and_neighbours<- data.table(expand.grid.alt2((coordinates[1] - raxial):(coordinates[1] + raxia
                                                      (coordinates[2] - raxial):(coordinates[2] + raxia
  target<- data.table(row = coordinates[1], col = coordinates[2])

  neighbours<- data.table(fsetdiff(target_and_neighbours, target))

  return(neighbours)
}

## Function to determine the maximum travelling range of the agent
expand.grid.alt2 <- function(seq1,seq2) {
  cbind(row=rep.int(seq1, length(seq2)), col=rep(seq2, each=length(seq1)))
}
get_range2 <- function(coordinates, max_range) {

  coordinates<- as.vector(as.matrix(coordinates))## ensure the coordinates are in the vector format

  data.frame(expand.grid.alt2((coordinates[1] - max_range):(coordinates[1] + max_range),
                              (coordinates[2] - max_range):(coordinates[2] + max_range))) %>%
    dplyr::filter(row > 0,
                  col > 0,
                  !(row == coordinates[1] & col == coordinates[2]))

}

## Function to determine the number of free cells where agents can occupy
get_freespots <- function(field_matrix){

  all_coordinates<- update_all(field_matrix)
  agent_coordinates<- update_agents(field_matrix)
```

```
  exposed_coordinates<- update_exposed(field_matrix)
  infected_coordinates<- update_infected(field_matrix)
  recovered_coordinates<- update_recovered(field_matrix)
  dead_coordinates<- update_dead(field_matrix)
  decomp_coordinates<- update_decomp(field_matrix)

  df.all<- data.table::data.table(all_coordinates)
  df.agent<- data.table::data.table(agent_coordinates)
  df.exposed<- data.table::data.table(exposed_coordinates)
  df.infected<- data.table::data.table(infected_coordinates)
  df.recovered<- data.table::data.table(recovered_coordinates)
  df.dead<- data.table::data.table(dead_coordinates)
  df.decomp<- data.table::data.table(decomp_coordinates)


  df<- data.table::fsetdiff(df.all, df.agent)
  df<- data.table::fsetdiff(df, df.exposed)
  df<- data.table::fsetdiff(df, df.infected)
  df<- data.table::fsetdiff(df, df.recovered)
  df<- data.table::fsetdiff(df, df.dead)
  df<- data.table::fsetdiff(df, df.decomp)

  df<- as.matrix(df)
  return(df)
}

### Function to determine the free-movable spaces within movement range
get_free_range2 <- function(coordinates, max_range, free_spots) {

  coordinates<- as.vector(as.matrix(coordinates))## ensure the coordinates are in the vector format

  movable_range<- data.table(expand.grid.alt2((coordinates[1] - max_range):(coordinates[1] + max_range)
                                              (coordinates[2] - max_range):(coordinates[2] + max_range))
  target<- data.table(row = coordinates[1], col = coordinates[2])

  movable_range2<- fsetdiff(movable_range, target)

  free_movable<- fintersect(movable_range2, data.table(free_spots))


  return(free_movable)

}

## Function to update all movable cells in the virtual background
update_all<- function(field_matrix){

  all_coordinates<- which(!is.na(field_matrix), arr.ind = T)

  return(all_coordinates)
}

## Function to update the agent locations in the virtual background
```

```r
update_agents<- function(field_matrix){

  agent_coordinates<- which(field_matrix>0.5, arr.ind = T)

  return(agent_coordinates)
}

## Function to update the susceptible agent locations in the area (value = 1)
update_susceptible<- function(field_matrix){

  susceptible_coordinates<- which(field_matrix>0.5 & field_matrix<2, arr.ind = T)

  return(susceptible_coordinates)
}

## Function to update the infected agent locations in the area (value = 2)
update_exposed<- function(field_matrix){

  exposed_coordinates<- which(field_matrix>1.5 & field_matrix<3, arr.ind=T)

  return(exposed_coordinates)
}

## Function to update the infected_acute agent locations in the area (value = 3.1 and 3.2)
update_infected<- function(field_matrix){

  infected_coordinates<- which(field_matrix>2.5 & field_matrix<4, arr.ind=T)

  return(infected_coordinates)
}

## Function to update the infected_acute agent locations in the area (value = 3.1)
update_infectedA<- function(field_matrix){

  infected_coordinates<- which(field_matrix>2.5 & field_matrix<3.2, arr.ind=T)

  return(infected_coordinates)
}

## Function to update the infected_chronic agent locations in the area (value = 3.2)
update_infectedC<- function(field_matrix){

  infected_coordinates<- which(field_matrix>3.1 & field_matrix<4, arr.ind=T)

  return(infected_coordinates)
}

## Function to update the infected agent locations in the area (value = 4)
update_recovered<- function(field_matrix){

  recovered_coordinates<- which(field_matrix>3.5 & field_matrix<4.5, arr.ind = T)

  return(recovered_coordinates)
```

```
}

## Function to update the dead agent locations in the area (value = 5)
update_dead<- function(field_matrix){

  dead_coordinates<- which(field_matrix>4.5 & field_matrix<5.5, arr.ind = T)

  return(dead_coordinates)
}

## Function to update the decomposed agent locations in the area (value = )
update_decomp<- function(field_matrix){

  decomp_coordinates<- which(field_matrix>5.5 & field_matrix<6.5, arr.ind = T)

  return(decomp_coordinates)
}

## Function to simulate agent movement (randomly occupy a free cell, may or may not move)
move_agents<- function(agentID, field_matrix, max_range, empty_matrix){

  free_spots<- get_freespots(field_matrix)
  agent_coordinates<- update_agents(field_matrix)
  # Check agent's status
  agent_stat<- field_matrix[agent_coordinates]

  ## Determine agent's current environment (i.e. forested or non-forested)
  agent_env<- empty_matrix[agent_coordinates]

    ## Determine if the target will move by "rolling a dice" rbinom; (Assumes that pigs will move more i
  decision<- rbinom(nrow(agent_coordinates), 1, (1-agent_env*6)*0.8)
  decision[agent_stat>4.5]<- 0 ## Dead and decomposed pigs cannot move
  decision[is.na(decision)]<- 0

  ## Movement of agent

    # Filter out agent who will stay put
  agent_move<- agent_coordinates[decision==1,]
  agent_stay<- agent_coordinates[decision==0,]

  #Status of agents who'll stay
  agent_stay_stat<- field_matrix[agent_stay]

  #Status of agents who'll be moving
  agent_move_stat<- field_matrix[agent_move]
  agent_move_env<- empty_matrix[agent_move]

  ## Determine the free space that each agent can move into
  agent_list<- split(data.table(agent_move), seq(1:nrow(agent_move)))
  agent_range_free<- lapply(agent_list, function(x) get_free_range2(x, max_range, free_spots))

  ## New coordinates of the agents that moved
  agent_move_new<- as.matrix(do.call(rbind, lapply(agent_range_free, function(x) dplyr::slice_sample(x,
```

```
  row.names(agent_move_new)<- NULL

  max_iter = 10
  iter = 0

   ### Check if there are any duplicated entries (i.e. two individuals moving into the same grid)
  while(any(duplicated(agent_move_new)) & iter < max_iter){
    agent_move_new[duplicated(agent_move_new),]<- as.matrix(do.call(rbind, lapply(agent_range_free[dupli
    iter = iter + 1
  }

  #if there's only one free grid with two agents, need to limit the movement of one agent at random
  agent_stuck<- as.integer(row.names(do.call(rbind, lapply(lapply(agent_range_free[duplicated(agent_move
  agent_move_new[agent_stuck,]<- agent_move[agent_stuck,]

  ## New coordinates of all agents
  agent_coordinates_new<- rbind(agent_move_new, agent_stay)

  ## Update field_matrix when agent leaves current position
  field_matrix[agent_move]<- agent_move_env
  field_matrix[agent_move_new]<- agent_move_stat

  agent_stat_updated<- field_matrix[agent_coordinates_new]
  agent_coordinates_old<- rbind(agent_move, agent_stay)

  agentID_new<- agentID[match(paste(agent_coordinates_old[,1], agent_coordinates_old[,2], sep="_"), past

  agentID_updated<- cbind(agent_coordinates_new, ID = agentID_new, status = agent_stat_updated)

  return(list(field_matrix, agentID_updated))

}

## Function to simulate an infectious agent (Infected, Carrier, or Dead) spreading it's disease to neig
exposed_agents<- function(agentID, agent_ID_matrix, field_matrix, transmit_p, raxial, latent_period, re
  library(dplyr)

  all_coordinates<- update_all(field_matrix)
  agent_coordinates<- update_agents(field_matrix)
  susceptible_coordinates<- update_susceptible(field_matrix)
  exposed_coordinates<- update_exposed(field_matrix)
  infected_coordinates<- update_infected(field_matrix)
  recovered_coordinates<- update_recovered(field_matrix)
  dead_coordinates<- update_dead(field_matrix)

  ## Check if there are any infected or carrier agents or dead carcass for exposure
  infected_flag<- nrow(infected_coordinates)>0
  recovered_flag<- nrow(recovered_coordinates)>0
  dead_flag<- nrow(dead_coordinates)>0

  latent_period_random = 0

  ## Latest coordinates and status of all agents
```

```
latest_matrix<-   last(agent_ID_matrix)

## Exposure, infection, and death history of all agents
history_data<- data.table(list.rbind(agent_ID_matrix))[,.(N = .N), .(status, ID)]


### Infection history
infect_history<- na.omit(history_data[history_data$status>2.5 & history_data$status < 4,])
infected_history_flag<- nrow(infect_history[infect_history$N > latent_period_random,])>0

recover_history<- na.omit(history_data[history_data$status>3.5 & history_data$status < 4.5,])
recover_history_flag<- nrow(recover_history[recover_history$N < recover_infect_period,])>0


## Will only run this segment only when there are infected, carrier (recovered) or dead agents for tr
## Contact with infected agent
if(infected_flag==TRUE){

  # Infected, Recovered and Dead individuals are referred to as "infectious" individuals. But need to
  # Infected agents

  ## Infection history
  infect_history<- na.omit(history_data[history_data$status>2.5 & history_data$status < 4,])
  ## Infection history that past latent period
  infect_history_pastlatent<- infect_history[infect_history$N > latent_period_random,]
  infect_history_pastlatent$row<- as.vector(latest_matrix[latest_matrix[,"ID"] %in% infect_history_pas
  infect_history_pastlatent$col<- as.vector(latest_matrix[latest_matrix[,"ID"] %in% infect_history_pas
  infect_coordinates<- infect_history_pastlatent[,c("row","col")]

  # Susceptible individuals transmitted by Infected agents
  susceptible_infected_coordinates<- data.table::fsetdiff(data.table(susceptible_coordinates), data.ta

  infect_list<- split(data.table(infect_history_pastlatent[,c("row","col")]), seq(1:nrow(infect_histor

  expose_infected_range<- lapply(infect_list, function(x) get_neighbors(x, raxial))
  susceptible_infected_exposed<- lapply(expose_infected_range, function(x) data.table::fintersect(x, c
  susceptible_infected_exposed<- do.call(rbind, susceptible_infected_exposed)
  susceptible_infected_exposed<- unique(susceptible_infected_exposed)
  row.names(susceptible_infected_exposed)<- NULL

  # Transmission rate of infected agent
  transmit_p_infected<- transmit_p[1]

  ## Determine if the Susceptible targets will transit to Exposed through the transmission likelihood
  exposed_infected_likelihood<- rbinom(nrow(susceptible_infected_exposed),1, transmit_p_infected)
  exposed_infected_likelihood
  ##### UPDATE AGENT STATUS BASED ON THE TRANSMISSION LIKELIHOOD AND INFECTION HISTORY (I.E. PAST LAT

  ## Determine the newly exposed agents due to infection from Infected agents
  susceptible_infected_exposed<- as.matrix(susceptible_infected_exposed)

  ## Update field_matrix when agent becomes exposed
  ## Update the agent's exposure status
```

```
    field_matrix[as.matrix(susceptible_infected_exposed)]<- field_matrix[as.matrix(susceptible_infected_

  }

  ## Contact with recovered agent (carrier and is still within infectious stage)
  if(recovered_flag==TRUE){

    # Infected, Recovered and Dead individuals are referred to as "infectious" individuals. But need to
    # Infected agents

    ## Infection history
    recover_history<- na.omit(history_data[history_data$status>3.5 & history_data$status < 4.5,])

    ## recover history and is still within the recover-infectious period
    recover_history_within<- recover_history[recover_history$N < recover_infect_period,]
    recover_history_within$row<- latest_matrix[latest_matrix[,3] %in% recover_history_within$ID, "row"]
    recover_history_within$col<- latest_matrix[latest_matrix[,3] %in% recover_history_within$ID, "col"]
    recover_coordinates<- recover_history_within[,c("row","col")]

    # Susceptible individuals transmitted by Infected agents
    susceptible_recover_coordinates<- data.table::fsetdiff(data.table(susceptible_coordinates), data.ta

    recover_list<- split(data.table(recover_history_within[,c("row","col")]), seq(1:nrow(recover_histor

    expose_recovered_range<- lapply(recover_list, function(x) get_neighbors(x, raxial))
    susceptible_recovered_exposed<- lapply(expose_recovered_range, function(x) data.table::fintersect(x
    susceptible_recovered_exposed<- do.call(rbind, susceptible_recovered_exposed)
    susceptible_recovered_exposed<- unique(susceptible_recovered_exposed)
    row.names(susceptible_recovered_exposed)<- NULL

    # Transmission rate of recovered agent
    transmit_p_recovered<- transmit_p[2]

    ## Determine if the Susceptible targets will transit to Exposed through the transmission likelihood
    exposed_recovered_likelihood<- rbinom(nrow(susceptible_recovered_exposed),1, transmit_p_recovered)

    ##### UPDATE AGENT STATUS BASED ON THE TRANSMISSION LIKELIHOOD AND RECOVERY HISTORY (I.E. WITHIN IN

    ## Determine the newly exposed agents due to recoverion from recovered agents
    susceptible_recovered_exposed<- as.matrix(susceptible_recovered_exposed)

    ## Update field_matrix when agent becomes exposed
    ## Update the agent's exposure status
    field_matrix[as.matrix(susceptible_recovered_exposed)]<- field_matrix[as.matrix(susceptible_recover

  }

  ## Contact with dead carcass
  if(dead_flag==TRUE ){


    # Dead agents
    # Susceptible individuals transmitted by Dead agents
```

```
    susceptible_dead_coordinates<- data.table::fsetdiff(data.table(susceptible_coordinates), data.table

    dead_list<- split(as.data.frame(dead_coordinates), seq(1:nrow(dead_coordinates)))

    ## identify the susceptible_dead neighbours of target in range (raxial) which will be exposed
    expose_dead_range<- lapply(dead_list, function(x) get_neighbors(x, raxial))
    susceptible_dead_exposed<- lapply(expose_dead_range, function(x) data.table::fintersect(x, data.tabl
    susceptible_dead_exposed<- do.call(rbind, susceptible_dead_exposed)
    susceptible_dead_exposed<- unique(susceptible_dead_exposed)
    row.names(susceptible_dead_exposed)<- NULL


    ## Transmission rate of dead agents (immediately becomes exposed)
    transmit_p_dead<- transmit_p[3]


    ## Determine if the Susceptible targets will transit to Exposed through the transmission likelihood
    exposed_dead_likelihood<- rbinom(nrow(susceptible_dead_exposed),1, transmit_p_dead)

    ## Determine the newly exposed agents due to recoverion from recovered agents
    susceptible_dead_exposed<- as.matrix(susceptible_dead_exposed)

    ## Update field_matrix when agent becomes exposed
    ## Update the agent's exposure status
    field_matrix[as.matrix(susceptible_dead_exposed)]<- field_matrix[as.matrix(susceptible_dead_exposed


  }



  ### Update status in agentID file (tracking the agent's status and position)

  agentID_coor<- paste(agentID[,1], agentID[,2], sep="_")
  agent_coor<- paste(agent_coordinates[,1], agent_coordinates[,2], sep="_")

  agentID[,4]<- field_matrix[agent_coordinates][match(agentID_coor, agent_coor)]

  ### Update field_matrix and agentID status
  return(list(field_matrix, agentID))
}

## Function to simulate the transition from Exposed to Infected_acute and Infected_chronic after the inc
infect_agents<- function(agentID, agent_ID_matrix, field_matrix, raxial, incubation_period, severe_p, t
  library(dplyr)

  all_coordinates<- update_all(field_matrix)
  agent_coordinates<- update_agents(field_matrix)
  exposed_coordinates<- update_exposed(field_matrix)

  ## Check if there are any Exposed agents to transit to Infected agents after the incubation period
  exposed_flag<- nrow(exposed_coordinates)>0
```

```
  ## Exposure, infection, and death history of all agents
  history_data<- data.table(list.rbind(agent_ID_matrix))[,.(N = .N), .(status, ID)]

  ## Randomise incubation period
  incubation_period_random<- rpois(1,  incubation_period)

  if(exposed_flag==TRUE & t_count > incubation_period_random){

    ## Exposure history
    expose_history<- na.omit(history_data[history_data$status>1.5 & history_data$status <2.5,])

    ## Exposure history that past incubation period and becomes infectious
    expose_history_postincubate<- expose_history[expose_history$N > incubation_period_random,]
    expose_history_postincubate$row<- agentID[match(expose_history_postincubate$ID,agentID[,3]),"row"]
    expose_history_postincubate$col<- agentID[match(expose_history_postincubate$ID,agentID[,3]),"col"]
    expose_infected_coordinates<- expose_history_postincubate[,c("row","col")]

    expose_infected_coordinates<-  fintersect(data.table(exposed_coordinates), data.table(expose_infecte


    ## Determine probability of that an exposed agent will become infectious (infectA and infectC) base
    infectA_likelihood<- rbinom(nrow(expose_infected_coordinates), 1, severe_p)
    infectC_likelihood<- abs(1-infectA_likelihood)*1.2
    infect_likelihood<- infectA_likelihood + infectC_likelihood

    ## Update field_matrix
    ## Agent becomes exposed
    ## Update the agent's exposure status
    field_matrix[as.matrix(expose_infected_coordinates)]<- field_matrix[as.matrix(expose_infected_coordi

  }


  ### Update status in agentID file

  agentID_coor<- paste(agentID[,1], agentID[,2], sep="_")
  agent_coor<- paste(agent_coordinates[,1], agent_coordinates[,2], sep="_")
  agentID[,4]<- field_matrix[agent_coordinates][match(agentID_coor, agent_coor)]

  ### Update field_matrix and agentID status
  return(list(field_matrix, agentID))
}

## Function to simulate the recovery of an infected agent (Can only commence when t_count > recovery pe
recover_agents<- function(agentID, agent_ID_matrix, field_matrix, recover_p, inf_hist_matrix, t_count, i

  infected_coordinates<- update_infected(field_matrix)
  agent_coordinates<- update_agents(field_matrix)
  recovered_coordinates<- update_recovered(field_matrix)


  ## Check if there are any infected agents
  infected_flag<- nrow(infected_coordinates)>0
```

```
recovered_flag<- nrow(recovered_coordinates)>0


## Exposure, infection, recovery, and death history of all agents
history_data<- data.table(list.rbind(agent_ID_matrix))[,.(N = .N), .(status, ID)]

## Randomise recovery period (can consider for exposure/infection history)
recovery_period_random<- rpois(1, recovery_period)

if(infected_flag==TRUE & t_count > recovery_period_random){

  ## Exposure history
  infect_history<- na.omit(history_data[history_data$status>2.5 & history_data$status <3.5,])

  ## Exposure history that past incubation period and becomes infectious
  infect_history_pastrecover<- infect_history[infect_history$N >  recovery_period_random,]
  infect_history_pastrecover$row<- agentID[match(infect_history_pastrecover$ID,agentID[,3]),"row"]
  infect_history_pastrecover$col<- agentID[match(infect_history_pastrecover$ID,agentID[,3]),"col"]
  infect_history_coordinates<- infect_history_pastrecover[,c("row","col")]
  infect_history_coordinates<-  fintersect(data.table(infected_coordinates), data.table(infect_history

  ## Determine probability of that an infected agent will recover based on infection history and reco
  recover_likelihood<- rbinom(nrow(infect_history_coordinates), 1, recover_p)


  ## Update field_matrix
  ## Infected agent recovers
  ## Update the agent's exposure status
  field_matrix[as.matrix(infect_history_coordinates)]<- field_matrix[as.matrix(infect_history_coordina
}


## Randomise immune period (for recovered agents to revert back to susceptible agents)
immune_period_random<- rpois(1, immune_period)

if(recovered_flag==TRUE){

  ## Exposure history
  recover_history<- na.omit(history_data[history_data$status>3.5 & history_data$status <4.5,])

  ## Recover history that past incubation period and becomes recoverious
  recover_history_pastrecover<- recover_history[recover_history$N > immune_period_random,]
  recover_history_pastrecover$row<- agentID[match(recover_history_pastrecover$ID,agentID[,3]),"row"]
  recover_history_pastrecover$col<- agentID[match(recover_history_pastrecover$ID,agentID[,3]),"col"]
  recover_history_coordinates<- recover_history_pastrecover[,c("row","col")]
  recover_history_coordinates<-  fintersect(data.table(recovered_coordinates), data.table(recover_his

  ## Determine probability of that an infected agent will recover based on infection history and reco
  susceptible_likelihood<- rbinom(nrow(recover_history_coordinates), 1, 1)

  ## Update field_matrix
  ## Infected agent recovers
  ## Update the agent's exposure status
```

```r
    field_matrix[as.matrix(recover_history_coordinates)] <- susceptible_likelihood

  }


  agentID_coor<- paste(agentID[,1], agentID[,2], sep="_")
  agent_coor<- paste(agent_coordinates[,1], agent_coordinates[,2], sep="_")
  field_matrix[agent_coordinates][match(agentID_coor, agent_coor)]
  agentID[,4]<- field_matrix[agent_coordinates][match(agentID_coor, agent_coor)]

  ### Update field_matrix and agentID status
  return(list(field_matrix, agentID))
}

## Function to simulate the death of an infected agent (Can only commence when t_count > death period; 
dead_agents<- function(agentID, agent_ID_matrix, field_matrix, death_p, inf_hist_matrix, t_count, death

  all_coordinates<- update_all(field_matrix)
  agent_coordinates<- update_agents(field_matrix)
  infected_coordinates<- update_infected(field_matrix)
  infectedA_coordinates<- update_infectedA(field_matrix)
  infectedC_coordinates<- update_infectedC(field_matrix)

  ### Death rates of acute and chronic infections
  death_p_acute<- death_p[1]
  death_p_chronic<- death_p[2]

  ### Randomise the death period
  death_period_acute<- rpois(1, death_period[1])
  death_period_chronic<- rpois(1, death_period[2])

  ## Check if there are any infected agents that will die
  infected_flag<- nrow(infected_coordinates)>0


  ## Exposure, infection, recovery, and death history of all agents
  history_data<- data.table(list.rbind(agent_ID_matrix))[,.(N = .N), .(status, ID)]


  #### ACUTE CASES
  ## To only run this segment only when there are infected agents for recovery
  if(infected_flag==TRUE){

    ## Acute infection history
    infectA_history<- na.omit(history_data[history_data$status>2.5 & history_data$status <3.3,])

    ## Acute infection history that past incubation period and becomes infectious
    infectA_history_pastdeath<- infectA_history[infectA_history$N >  death_period_acute,]
    infectA_history_pastdeath$row<- agentID[match(infectA_history_pastdeath$ID,agentID[,3]),"row"]
    infectA_history_pastdeath$col<- agentID[match(infectA_history_pastdeath$ID,agentID[,3]),"col"]
    infectA_history_coordinates<- infectA_history_pastdeath[,c("row","col")]
    infectA_history_coordinates<-  fintersect(data.table(infectedA_coordinates), data.table(infectA_his
```

```
    ## Chronic infection history
    infectC_history<- na.omit(history_data[history_data$status>3.2 & history_data$status <3.5,])

    ## Chronic infection history that past incubation period and becomes infectious
    infectC_history_pastdeath<- infectC_history[infectC_history$N >  death_period_acute,]
    infectC_history_pastdeath$row<- agentID[match(infectC_history_pastdeath$ID,agentID[,3]),"row"]
    infectC_history_pastdeath$col<- agentID[match(infectC_history_pastdeath$ID,agentID[,3]),"col"]
    infectC_history_coordinates<- infectC_history_pastdeath[,c("row","col")]
    infectC_history_coordinates<-  fintersect(data.table(infectedC_coordinates), data.table(infectC_his

    ## Determine probability of that an infected agent will recover based on infection history and reco
    infectA_death_likelihood<- rbinom(nrow(infectA_history_coordinates), 1, death_p_acute)
    infectC_death_likelihood<- rbinom(nrow(infectC_history_coordinates), 1, death_p_chronic)


    ## Update field_matrix
    ## Infected agent dies
    ## Update the agent's death status
    field_matrix[as.matrix(infectA_history_coordinates)]<- field_matrix[as.matrix(infectA_history_coordi
    field_matrix[as.matrix(infectC_history_coordinates)]<- field_matrix[as.matrix(infectC_history_coordi

  }



  agentID_coor<- paste(agentID[,1], agentID[,2], sep="_")
  agent_coor<- paste(agent_coordinates[,1], agent_coordinates[,2], sep="_")
  field_matrix[agent_coordinates][match(agentID_coor, agent_coor)]
  agentID[,4]<- field_matrix[agent_coordinates][match(agentID_coor, agent_coor)]

  ### Update field_matrix and agentID status
  return(list(field_matrix, agentID))
}

## Function to simulate the decomposition of an infected carcass (Can only commence when t_count > deco
decomp_agents<- function(agentID, agent_ID_matrix, field_matrix, dead_hist_matrix, t_count, decomp_peri

  all_coordinates<- update_all(field_matrix)
  agent_coordinates<- update_agents(field_matrix)
  dead_coordinates<- update_dead(field_matrix)


  ## Check if there are any dead agents
  dead_flag<- nrow(dead_coordinates)>0


  ## Exposure, infection, recovery, and death history of all agents
  history_data<- data.table(list.rbind(agent_ID_matrix))[,.(N = .N), .(status, ID)]


  ## Randomise the decomposition period of carcass
  decomp_period_random<- rpois(1, decomp_period)
```

```
  ## To only run this segment only when there are infected agents for recovery
  if(dead_flag==TRUE){

    ## Infection history
    dead_history<- na.omit(history_data[history_data$status>4.5 & history_data$status <5.5,])

    ## Infection history that past incubation period and becomes infectious
    dead_history_pastdecomp<- dead_history[dead_history$N >  decomp_period_random,]
    dead_history_pastdecomp$row<- agentID[match(dead_history_pastdecomp$ID,agentID[,3]),"row"]
    dead_history_pastdecomp$col<- agentID[match(dead_history_pastdecomp$ID,agentID[,3]),"col"]
    dead_history_coordinates<- dead_history_pastdecomp[,c("row","col")]
    dead_history_coordinates<-  fintersect(data.table(dead_coordinates), data.table(dead_history_coordi

    ## Determine probability of decomposition based on death history
    prob_decomp<- 0.5
    decomp_likelihood<- rbinom(nrow(dead_history_coordinates), 1, prob_decomp)


    ## Update field_matrix
    ## Dead agent decomposes
    ## Update the agent's status
    field_matrix[as.matrix(dead_history_coordinates)]<- field_matrix[as.matrix(dead_history_coordinates)


  }


  agentID_coor<- paste(agentID[,1], agentID[,2], sep="_")
  agent_coor<- paste(agent_coordinates[,1], agent_coordinates[,2], sep="_")
  field_matrix[agent_coordinates][match(agentID_coor, agent_coor)]
  agentID[,4]<- field_matrix[agent_coordinates][match(agentID_coor, agent_coor)]

  ### Update field_matrix and agentID status
  return(list(field_matrix, agentID))
}
```
