# Supplementary material for: Modeling Singapore's First African Swine Fever Outbreak in Wild Boar Populations
Source: Transbound Emerg Dis. 2024 Aug 26;2024:5546893. doi: 10.1155/2024/5546893 (PMC12016949; doi:10.1155/2024/5546893)
Supplement: Supplementary 6 — Example code for the agent-based model and simulation. [file 5546893.f6.pdf]

Example code for ABM

Rayson Lim

2023-08-31

Example code for the agent-based simulation of the ASF transmission among wild boars

To understand the African Swine Fever transmission dynamics in Singapore, an agent-based spatio-temporal model was designed to mechanistically model the wild boar's movement and their interactions with the landscape for ASF transmission. This protocol is an example to demonstrate how the agent's movement can be influenced by the landscape, which affects the disease transmission dynamics. The disease transmission is modified from the classical epidemiological compartmental model (SIR) to include carcass-mediated and covalescent transmission pathways.

Loading libraries and source functions

```
#Set the work directory to where the source and code files are saved. Load the libraries and source fun

# load libraries
  libs <- c(
    "tidyr","raster","rlist","data.table", "ggplot2", "cowplot"
  )

  invisible(lapply(libs, library, character.only = T))
```

```
## Loading required package: sp

## The legacy packages maptools, rgdal, and rgeos, underpinning the sp package,
## which was just loaded, will retire in October 2023.
## Please refer to R-spatial evolution reports for details, especially
## https://r-spatial.org/r/2023/05/15/evolution4.html.
## It may be desirable to make the sf package available;
## package maintainers should consider adding sf to Suggests:.
## The sp package is now running under evolution status 2
##      (status 2 uses the sf package in place of rgdal)

##
## Attaching package: 'data.table'

## The following object is masked from 'package:raster':
##
##     shift
```

```r
# Load source functions for agent based model
  source("S2. Source functions for ABM.R")
```

Setting the virtual background

Customise the virtual background for the simulation where 0 = traversable area, 0.1 = forests, and NA = non-traversable areas.

```r
bkgrd<- matrix(sample(c(0, 0.1, 0.2), 2500, replace = T, prob = c(0.6, 0.3, 0.1)), ncol = 50, nrow = 50)
bkgrd<- raster(bkgrd)
bkgrd[which(values(bkgrd)==0.2)]<- NA #Areas where agents cannot traverse

plot(bkgrd)
```

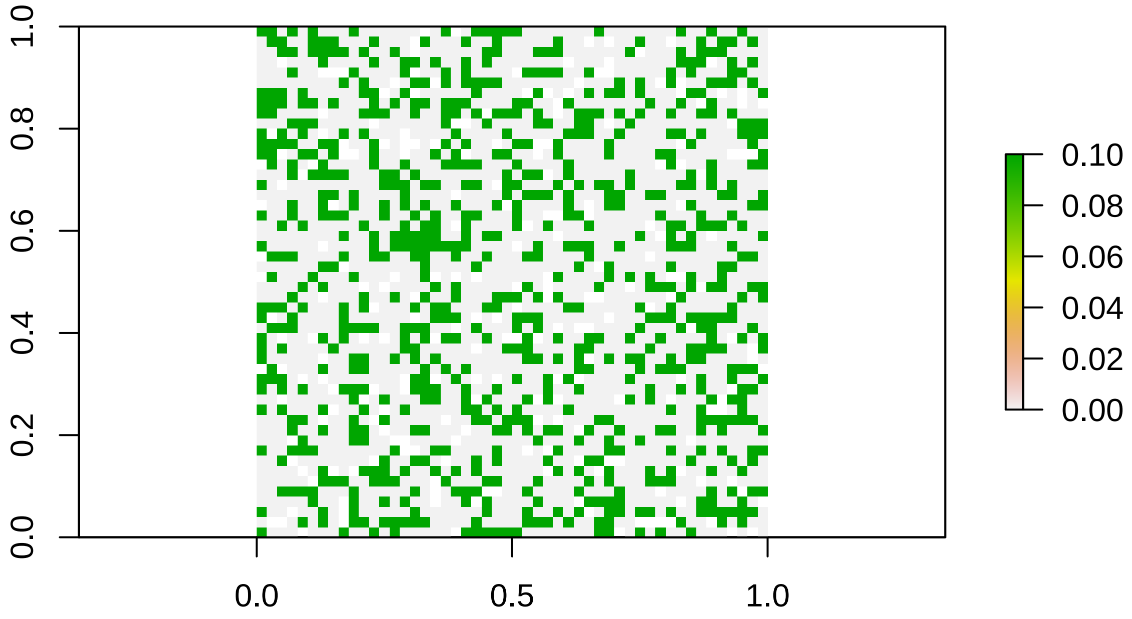

Assign agents

Randomly position agents (N = 50; value = 1) into the virtual layer and randomly choose one of them as infected (value = 3).

```r
# Randomly assign agents into virtual area
N = 50
agents<- sampleRandom(bkgrd, size = N,  sp=T, rowcol=T)
agents$layer<- 1

# Randomly choose one of the agent as infected (3)
```

```
agents$layer[sample.int(N, 1)]<- 3

# Rasterize and combined background with agents
ind.ras<- rasterize(agents, bkgrd, field = "layer", background=0)
area.ras<- ind.ras + bkgrd
plot(area.ras)
```

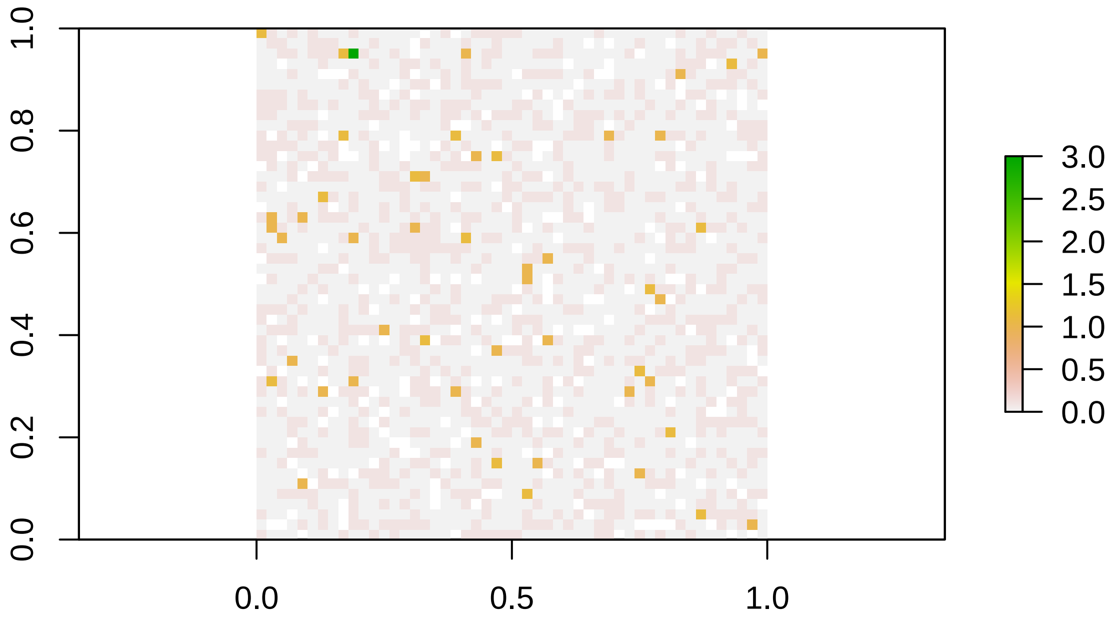

Setting the initial parameters for simulation

```
# Time_step to day ratio based on movement simulation (50 time steps = 1 day)
time_day<- 50

# Set initial parameters for the simulation
args<- as.vector(as.character(c(30*time_day, #1. Simulation time line (days)
                                1, #2. Initial number of infected agents
                                1, #3. Replicate number
                                0.685, #4. Transmission rate_infected
                                0.07, #5. Transmission rate_recovered
                                0.95, #6. Transmission rate_dead
                                0.05, #7. Recover rate
                                0.9, #8. Mortality rate of acute cases
                                0.45, #9. Mortality rate of chronic cases
                                7*time_day, #10. Latent period through oral transmission (Infected lo
```

```
                                  7*time_day, #11. Incubation period of Exposed individuals
                                  24*time_day, #12. Infectious period of recovered individuals (Carrier
                                  2*time_day, #13. Decomposition period (days)
                                  24*time_day, #14. Recovery period (days)
                                  24*time_day, #15. Immune period (days)
                                  4*time_day, #16. Time to death of acute cases (days)
                                  24*time_day, #17. Time to death of chronic cases (days)
                                  0.965 #18. Severity of disease (i.e. 90% acute, 10% chronic)
)))


# Replicate number
REP<- as.integer(args[3])

# Number of time step
t_step = as.integer(args[1])

# Maximum range which an individual can move
max_range = 1

# Transmission probability (from infected, carrier, and dead agents)
transmit_p_infected = as.numeric(args[4]) # Blome et al. 2012
transmit_p_recovered = as.numeric(args[5]) # Blome et al. 2012
transmit_p_dead = as.numeric(args[6]) # Blome et al. 2012
transmit_p = c(transmit_p_infected, transmit_p_recovered, transmit_p_dead)

# Severity of infection (i.e. likelihood to be acute infection relative to chronic (1-p))
severe_p = as.numeric(args[18])

# R-axial of Moore neighbourhood (i.e. disease spread radius; assumed to require close contact)
raxial = 1

# Recovery probability
recover_p = as.numeric(args[7])

# Recovery period
recovery_period = as.integer(args[14])

# Incubation period of exposed agents
incubation_period = as.integer(args[11])

# Immune period of recovered agents
immune_period = as.integer(args[15]) # assumed

# Death probability
death_p_acute = as.numeric(args[8]) # death likelihood after acute infection
death_p_chronic = as.numeric(args[9]) # death likelihood after chronic infection
death_p = c(death_p_acute, death_p_chronic)

# Death period (Time from infection to death)
death_period_acute = as.integer(args[16]) # time from infection to death for acute infections
death_period_chronic = as.integer(args[17]) # time from infection to death for chronic infections
death_period = c(death_period_acute, death_period_chronic)
```

```r
  # Decomposition period (Time from death; carcass/viral persistence )
  decomp_period = as.integer(args[13])

  # Latent period of infection (Time from contact to development of symptoms/considered as infected and
  latent_period = as.integer(args[10]) #days; through oral transmission

  # Infectious period for recovered agents (Carrier pigs remain infectious for a fixed duration)
  recover_infect_period<- as.integer(args[12]) #days

  # Coordinates of all movable spaces
  all_coor<- rowColFromCell(area.ras, which(!is.na(getValues(area.ras))))
  all_cell<- cellFromRowCol(area.ras, all_coor[,1], all_coor[,2])

  # Coordinates of all agents in the area (initial)
  ind_coor<- rowColFromCell(ind.ras, which(getValues(ind.ras)>0))
  ind_cell<- cellFromRowCol(area.ras, ind_coor[,1], ind_coor[,2])

  # Coordinates of infected agents in the area (initial)
  inf_cell<- which(values(ind.ras)==2)
  inf_coor<- rowColFromCell(area.ras, inf_cell)

  # Initial coordinates of exposed agents in the area (initial)
  exposed_coor<- data.frame(row=0, col=0)
  exposed_cell<-  cellFromRowCol(area.ras, exposed_coor[,1], exposed_coor[,2])

  # Initial coordinates of recovered agents in the area (initial)
  recover_coor<- data.frame(row=0, col=0)
  recover_cell<-  cellFromRowCol(area.ras, recover_coor[,1], recover_coor[,2])

  # Initial coordinates of dead agents in the area (initial)
  dead_coor<- data.frame(row=0, col=0)
  dead_cell<-  cellFromRowCol(area.ras, dead_coor[,1], dead_coor[,2])

  # Initial coordinates of decomposing agents in the area (initial)
  decomp_coor<- data.frame(row=0, col=0)
  decomp_cell<-  cellFromRowCol(area.ras, decomp_coor[,1], dead_coor[,2])

  # Combined all layers into a field matrix (background + individuals + infected)
  field_matrix<- as.matrix(area.ras)

  empty_matrix<- as.matrix(bkgrd)

  # Determine the initial background raster that is free for agents to move
  free_spots<- get_freespots(field_matrix)
```

Start the simulation

```r
# Initial time count
  t_count = 1

  # Storing agent's status information, raster output, and infection results as lists
```

```r
agent_ID_matrix<- list()
raster.output<- list()
result.output<- list()

# Initial position of agents and their ID
agentID<- cbind(which(field_matrix>0.5, arr.ind = T), ID = seq(1:nrow(which(field_matrix>0.5, arr.ind

  while (t_count<= t_step & length(which(field_matrix>1.5 & field_matrix<6))>0){

  tryCatch({
    ## Movement phase
    move.output<- move_agents(agentID, field_matrix, max_range, empty_matrix)
    field_matrix<- move.output[[1]]
    agentID<- move.output[[2]]
  }, error = function(e) return(paste0("Movement of agents",
                                       " caused the error: '", e, "'")))

  tryCatch({
    ## Start recording the exposure, infection, recovery histories and death positions of all agents
    agent_ID_matrix<- append(agent_ID_matrix, list(agentID))
  }, error = function(e) return(paste0("Recording infection history and death positions of agent",
                                       " caused the error: '", e, "'")))

  tryCatch({
    ## Exposure phase
    expose.output<- exposed_agents(agentID, agent_ID_matrix, field_matrix, transmit_p, raxial, latent
    field_matrix<- expose.output[[1]]
    agentID<- expose.output[[2]]
  }, error = function(e) return(paste0("Exposure of agents",
                                       " caused the error: '", e, "'")))

  tryCatch({
    ## Infection phase
    infect.output<- infect_agents(agentID, agent_ID_matrix, field_matrix, raxial, incubation_period, 
    field_matrix<- infect.output[[1]]
    agentID<- infect.output[[2]]
  }, error = function(e) return(paste0("Infection of agents",
                                       " caused the error: '", e, "'")))

  tryCatch({
    ## Recovery phase
    recover.output<- recover_agents(agentID, agent_ID_matrix, field_matrix, recover_p, inf_hist_matri
    field_matrix<- recover.output[[1]]
    agentID<- recover.output[[2]]
  }, error = function(e) return(paste0("Recovery of agents",
                                       " caused the error: '", e, "'")))

  tryCatch({
    ## Death phase
    death.output<- dead_agents(agentID, agent_ID_matrix, field_matrix, death_p, inf_hist_matrix, t_co
    field_matrix<- death.output[[1]]
    agentID<- death.output[[2]]
  }, error = function(e) return(paste0("Death of agents",
```

```
                                          " caused the error: '", e, "'")))

    tryCatch({
      ## Decomposition phase
      decomp.output<- decomp_agents(agentID, agent_ID_matrix, field_matrix, dead_hist_matrix, t_count, d
      field_matrix<- decomp.output[[1]]
      agentID<- decomp.output[[2]]
    }, error = function(e) return(paste0("Decomposition of agents",
                                          " caused the error: '", e, "'")))


     r1<- raster(field_matrix)
     e1<- extent(area.ras)
     extent(r1)<- e1
     c1<- crs(area.ras)
     crs(r1)<- c1


 # Plotting the raster at each 10-day interval
    if(t_count ==1 | t_count %% 500==0){
     r1<- raster(field_matrix)
     e1<- extent(area.ras)
     extent(r1)<- e1
     c1<- crs(area.ras)
     crs(r1)<- c1
     colvec<- c("grey","palegreen","palegreen","palegreen","palegreen","palegreen","blue","blue","blue"
     plot(r1, col=colvec[1:(5 + max(values(r1), na.rm=T)*4.5)], main = paste("Day = ", round(t_count/50
     Sys.sleep(0.5)

     }
    # Update the time step
    t_count = t_count + 1
    #print(paste(t_count, " of ", t_step))


    # Save the plot at daily intervals (i.e. 50 time-steps)
    if(t_count %% time_day==0){

      ### Compile the individual rasters at each time step as a list
      raster.output<- append(raster.output, r1)

      ### Save the number of agents who are infected/dead/decomposed and the simulation number to a data
      result.output.t<- list(data.table(time = t_count, expose_N = nrow(update_exposed(field_matrix)), i
      result.output<- append(result.output, result.output.t)
    }
  }
```

```
##
## Attaching package: 'dplyr'

## The following objects are masked from 'package:data.table':
##
##     between, first, last
```

```
## The following objects are masked from 'package:raster':
##
##     intersect, select, union

## The following objects are masked from 'package:stats':
##
##     filter, lag

## The following objects are masked from 'package:base':
##
##     intersect, setdiff, setequal, union
```

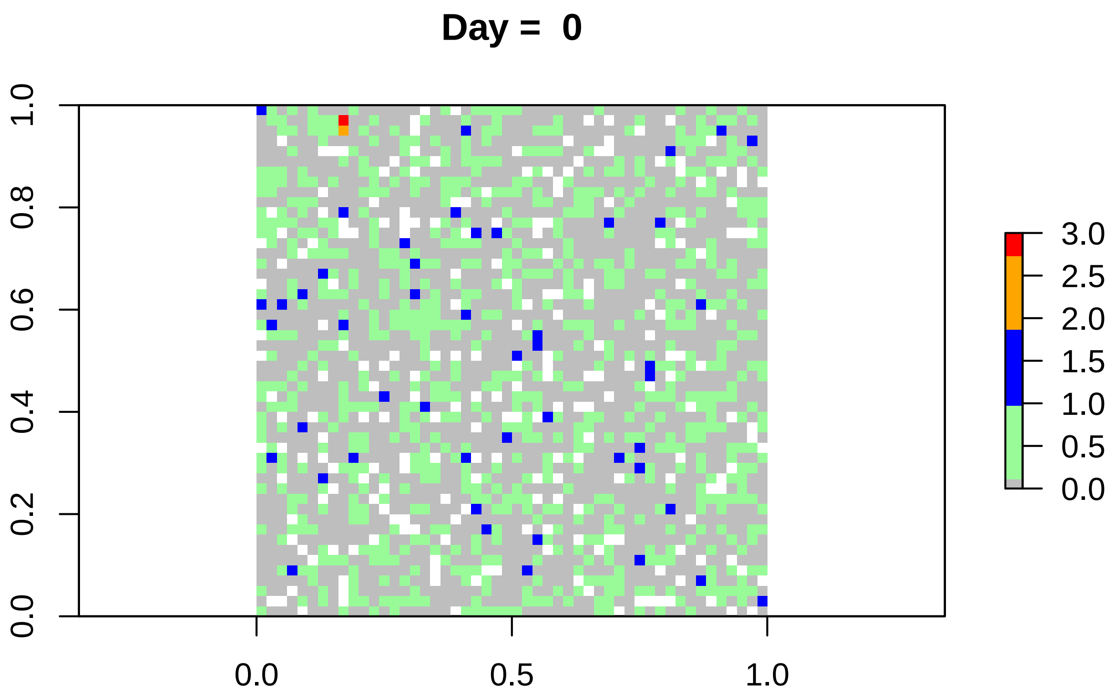

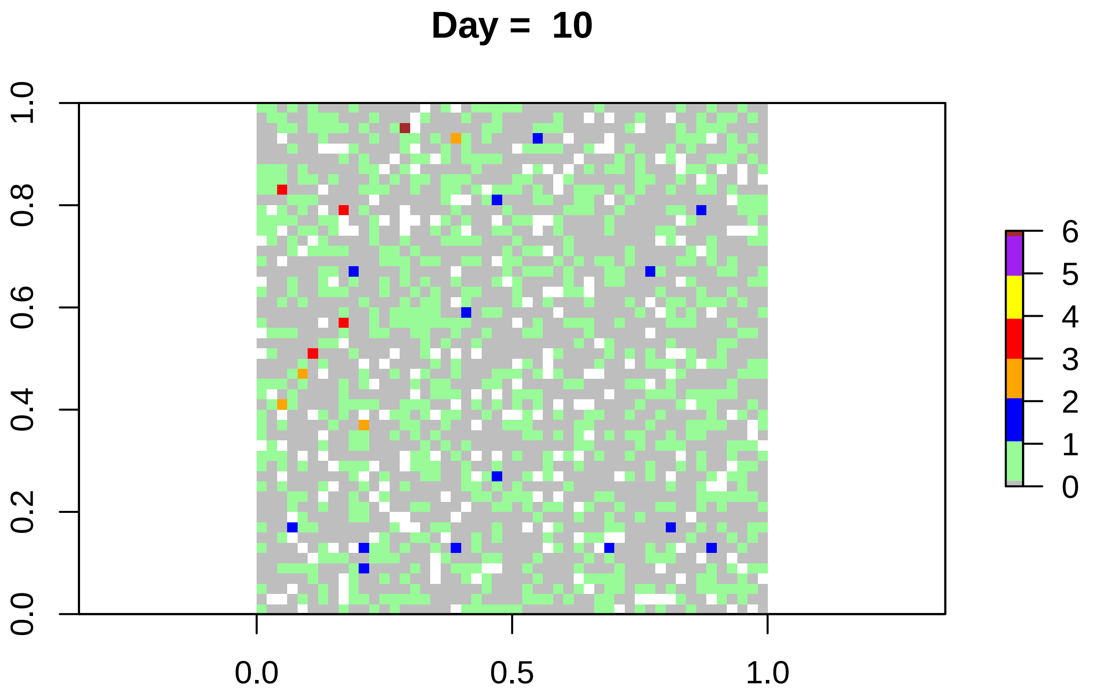
Day = 10
0.0
0.2
0.4
0.6
0.8
1.0
0.0
0.5
1.0
0
1
2
3
4
5
6

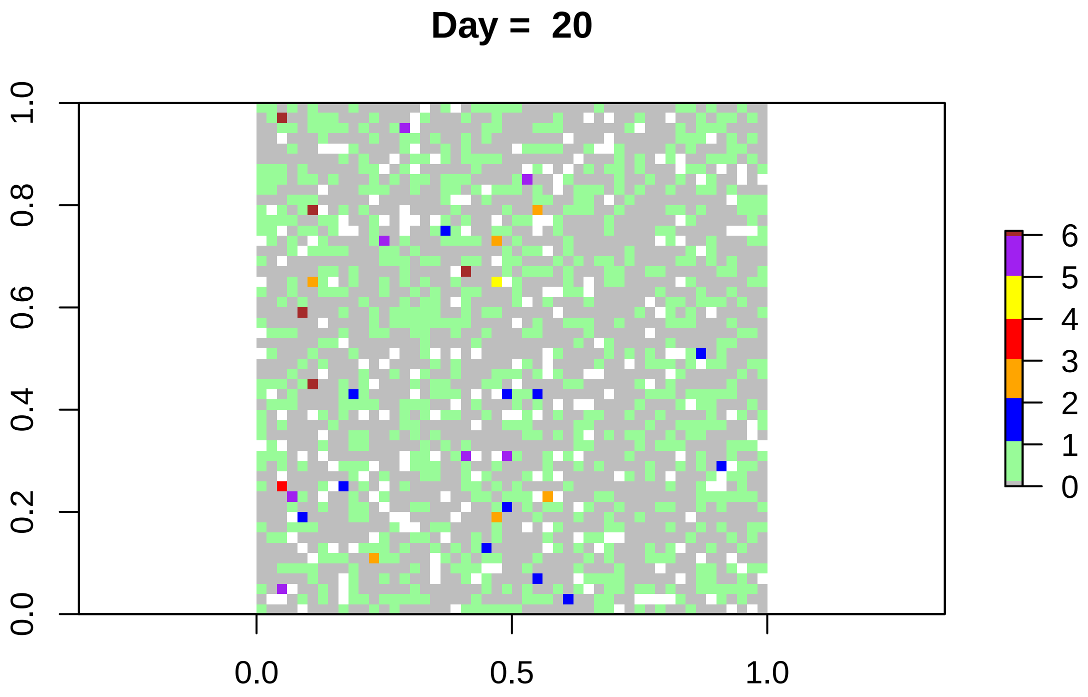

Day = 20
1.0
0.8
0.6
0.4
0.2
0.0
0.0
0.5
1.0
6
5
4
3
2
1
0

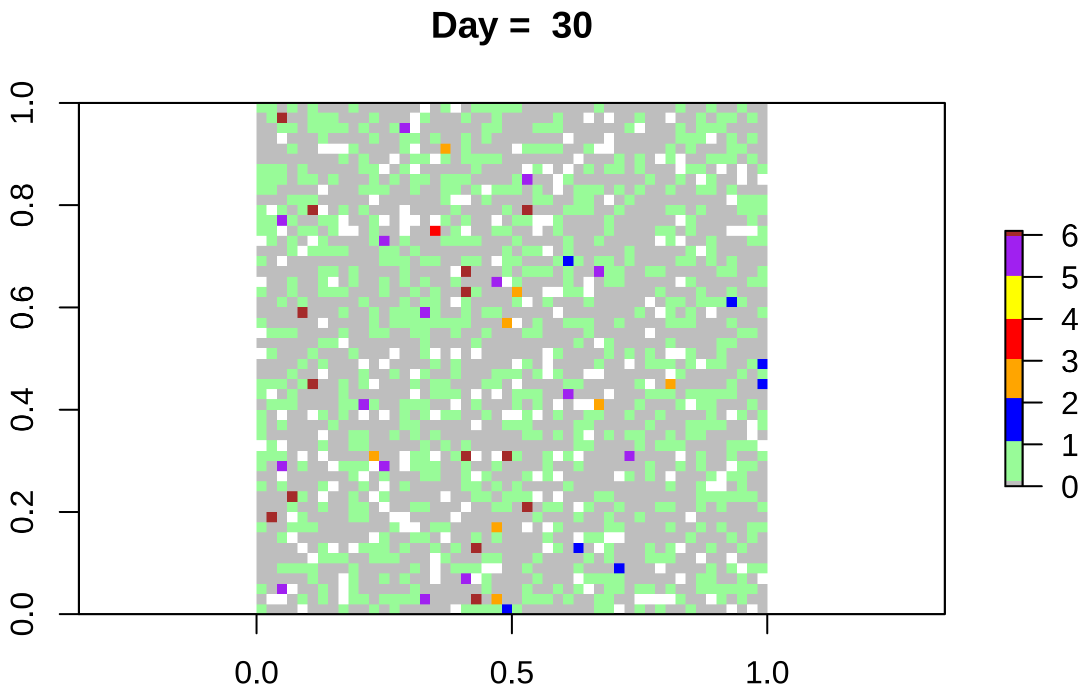


```
### Save the results
result.list<- list(result.output, raster.output, agent_ID_matrix)
```

Plot the epidemic curve

```
dat.out<- as.data.frame(do.call(rbind, lapply(result.list[[1]], function(x) do.call(cbind, x))))
plot_s<- ggplot(data=dat.out, aes(x=time/50,y=all_N-(expose_N+infect_N+recover_N+dead_N+decomp_N))) + ge
plot_e<- ggplot(data=dat.out, aes(x=time/50,y=expose_N)) + geom_line(alpha = 0.3, col = 'orange') + ther
plot_i<- ggplot(data=dat.out, aes(x=time/50,y=infect_N)) + geom_line(alpha = 0.3, col = 'red') + theme_l
plot_de<- ggplot(data=dat.out, aes(x=time/50,y=decomp_N)) + geom_line(alpha = 0.3, col = 'brown') + ther

ggdraw() +
  draw_plot(plot_s, x = 0, y = 1/2, width = 1/2, height = 1/2 )+
  draw_plot(plot_e, x = 0, y = 0, width = 1/2, height = 1/2 )+
  draw_plot(plot_i, x = 1/2, y = 1/2, width = 1/2, height = 1/2) +
  draw_plot(plot_de, x = 1/2, y = 0, width = 1/2, height = 1/2)
```

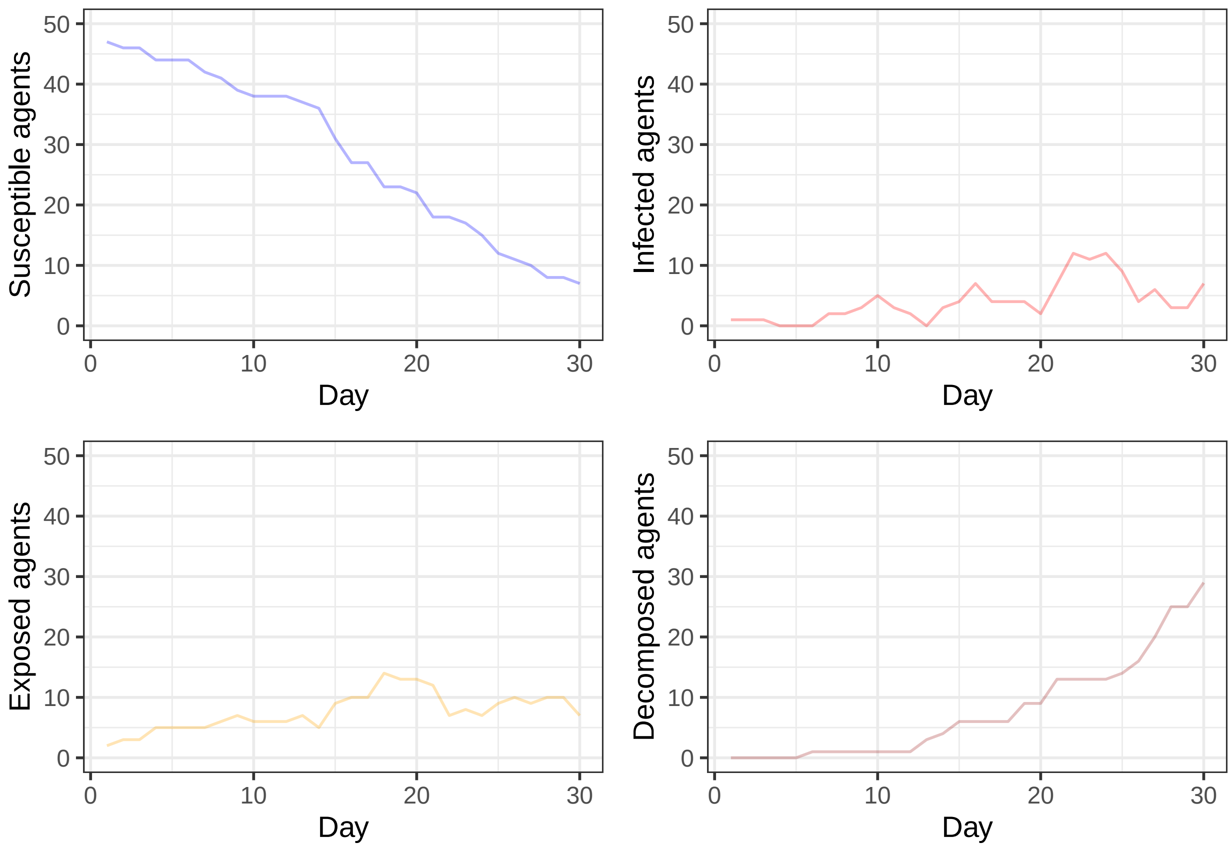
Susceptible agents
Infected agents
Exposed agents
Decomposed agents
Day
0
10
20
30
40
50
